# Effect of the Nature of Donor Atoms on the Thermodynamic, Kinetic and Relaxation Properties of Mn(II) Complexes Formed With Some Trisubstituted 12-Membered Macrocyclic Ligands

**DOI:** 10.3389/fchem.2018.00232

**Published:** 2018-08-13

**Authors:** Zoltán Garda, Enikő Molnár, Ferenc K. Kálmán, Richárd Botár, Viktória Nagy, Zsolt Baranyai, Ernő Brücher, Zoltán Kovács, Imre Tóth, Gyula Tircsó

**Affiliations:** ^1^Department of Inorganic and Analytical Chemistry, Faculty of Science and Technology, University of Debrecen, Debrecen, Hungary; ^2^Advanced Imaging Research Center, The University of Texas Southwestern Medical Center, Dallas, TX, United States

**Keywords:** Mn(II) complexes, contrast agents for MRI, stability, inertness, relaxivity

## Abstract

During the past few years increasing attention has been devoted to Mn(II) complexes as possible substitutes for Gd(III) complexes as contrast agents in MRI. Equilibrium (log *K*_MnL_ or pMn value), kinetic parameters (rates and half-lives of dissociation) and relaxivity of the Mn(II) complexes formed with 12-membered macrocyclic ligands were studied. The ligands were selected in a way to gain information on how the ligand rigidity, the nature of the donor atoms in the macrocycle (pyridine N, amine N, and etheric O atom), the nature of the pendant arms (carboxylates, phosphonates, primary, secondary and tertiary amides) affect the physicochemical parameters of the Mn(II) complexes. As expected, decreasing the denticity of DOTA (to afford DO3A) resulted in a drop in the stability and inertness of [Mn(DO3A)]^−^ compared to [Mn(DOTA)]^2−^. This decrease can be compensated partially by incorporating the fourth nitrogen atom into a pyridine ring (e.g., PCTA) or by replacement with an etheric oxygen atom (ODO3A). Moreover, the substitution of primary amides for acetates resulted in a noticeable drop in the stability constant (PC3AM^H^), but it increased as the primary amides (PC3AM^H^) were replaced by secondary (PC3AM^Gly^) or tertiary amide (PC3AM^Pip^) pendants. The inertness of the Mn(II) complexes behaved alike as the rates of acid catalyzed dissociation increased going from DOTA (*k*_1_ = 0.040 M^−1^s^−1^) to DO3A (*k*_1_ = 0.45 M^−1^s^−1^). However, the rates of acid catalyzed dissociation decreased from 0.112 M^−1^s^−1^ observed for the anionic Mn(II) complex of PCTA to 0.0107 M^−1^s^−1^ and 0.00458 M^−1^s^−1^ for the cationic Mn(II) complexes of PC3AM^H^ and PC3AM^Pip^ ligands, respectively. In spite of its lower denticity (as compared to DOTA) the sterically more hindered amide complex ([Mn(PC3AM^Pip^)]^2+^) displays surprisingly high conditional stability (pMn = 8.86 vs. pMn = 9.74 for [Mn(PCTA)]^−^) and excellent kinetic inertness. The substitution of phosphonates for the acetate pendant arms (DOTP and DO3P), however, resulted in a noticeable drop in the conditional stability as well as dissociation kinetic parameters of the corresponding Mn(II) complexes ([Mn(DOTP)]^6−^ and [Mn(DO3P)]^4−^) underlining that the phosphonate pedant should not be considered as a suitable building block for further ligand design while the tertiary amide moiety will likely have some implications in this respect in the future.

## Introduction

In the recent years, the research in the field of Mn(II) coordination chemistry has been intensified aiming to develop Mn(II) complexes suitable for *in vivo* application as magnetic resonance imaging (MRI) contrast agents (CA) (Drahos et al., 2012; Gale et al., 2015; Forgács et al., 2016; Garda et al., 2016). Gd(III) complexes are used as CAs in millions of doses. These agents had been assumed to be safe, however, nephrogenic systemic fibrosis (NSF), a devastating disease discovered in the late 90s has pointed out that they can cause serious health problems in patients with severe renal impairment (Idee et al., 2006). Thus, the design of safer CAs for MRI might be achieved by replacing the paramagnetic metal center with one that is better tolerated by the living systems (e.g., essential metal ions like Mn(II) or Fe(II)). The Mn(II) complexes are considered to be safe alternatives to Gd(III) in MRI as Mn(II) is an essential metal ion and therefore, biological systems can efficiently control its homeostasis (Murakami et al., 1996; Aime et al., 2002; Balogh et al., 2007; Drahos et al., 2011; Kálmán and Tircsó, 2012; Gale et al., 2015; Garda et al., 2016). However, the lack of ligand-field stabilization in high spin Mn(II) complexes results in lower thermodynamic stability compared to other divalent essential metal and the trivalent Gd(III) complexes. In addition, most Mn(II) chelates are kinetically labile. Due to these factors, the development of safe Mn(II) MRI CAs for *in vivo* applications remains challenging. We have shown 6 years ago that only *trans-*CDTA (*trans-*CDTA = *trans*-1,2-diaminocyclohexane-*N,N,N*′*,N*′-tetraacetic acid) forms thermodynamically stable and kinetically inert Mn(II) chelate with acceptable relaxation enhancement out of several Mn(II) complexes of open-chain and AAZTA (AAZTA = 6-amino-6-methylperhydro-1,4-diazepine tetraacetic acid) ligands (Kálmán and Tircsó, 2012). This observation inspired the development of new open-chain ligands for Mn(II) complexation such as PyC3A, PhDTA, BEDIK (PyC3A = *N*-picolyl-*N,N*′*,N*′-*trans*-1,2-cyclohexylenediamine-triacetate, PhDTA = *ortho*-phenylenediamine-*N,N,N*′*,N*′-tetraacetic acid, BEDIK = 2-(aminomethyl)aniline-*N,N,N*′*,N*′-tetraacetic acid), derivatives of 2,6-bis-aminometyl pyridine and various open-chain ligands incorporating the picolinate moiety (Su et al., 2012; Forgács et al., 2015, 2017; Gale et al., 2015; Phukan et al., 2015; Wu et al., 2015; Póta et al., 2018) as well as redox activated Mn(II)-based MRI CA candidates (Loving et al., 2013). The design and synthesis of new bifunctional chelators (BFCs) derived from *trans-*1,2-diaminocyclohexane for targeted imaging applications has also been reported (Gale et al., 2015; Vanasschen et al., 2017).

Macrocyclic ligands have also been screened with the aim of finding a suitable macrocyclic platform for Mn(II) complexation. The investigated macrocyclic ligands were mostly the acetate, rarely the phosphonate and phosphinate derivatives of tacn (tacn = 1,4,7,-triazacyclononane), cyclen and pyclen; efforts were also devoted to the investigation of some rigid pyridine-based 15-membered and other macrocyclic complexes formed with 9-, 12-, 14-, and 15-membered macrocyclic ligands (Dees et al., 2007; Drahos et al., 2010, 2011, 2012; Molnar et al., 2014; Forgács et al., 2016, 2017; Garda et al., 2016). The main goal of these studies was the development of a ligand platform that would allow a solvent molecule to be coordinated in the inner coordination sphere of the Mn(II) complexes, which is necessary to achieve appropriate relaxation enhancement. This was achieved in most cases; actually, some of the chelates, such as Mn(II) complexes of the rigid 15-PyaneN_5_ (3,6,9,12,18-pentaazabicyclo[12.3.1]octadeca-1(18),14,16-triene) and 15-PyaneN_3_O_2_ (3,12,18-Triaaza-6,9-dioxabicyclo[12.3.1]octadeca-1(18),14,16-triene) was found to have not just one, but two bound water molecules. However, most of these complexes were kinetically too labile for *in vivo* applications. The only structures that represented an acceptable compromise between the apparently contradictory requirements (thermodynamic and redox stability/inertness/relaxivity) were the Mn(II) chelates of the *cis*-DO2A and the 15-PyaneN_3_O_2_ ligands (Dees et al., 2007; Drahos et al., 2010; Garda et al., 2016). The kinetic properties improved by the replacement of two carboxylate moieties with dimethylamide metal binding units (Forgács et al., 2016). The results obtained in our laboratory by studying monopicolinates of 9-, 12- and 14-membered macrocyclic ligands have also indicated that the best kinetic data were obtained for the Mn(II) complex of a 12-membered macrocyclic derivative, but further ligand optimization is required to identify the best candidates for *in vivo* applications (Molnar et al., 2014).

**Figure 1:**
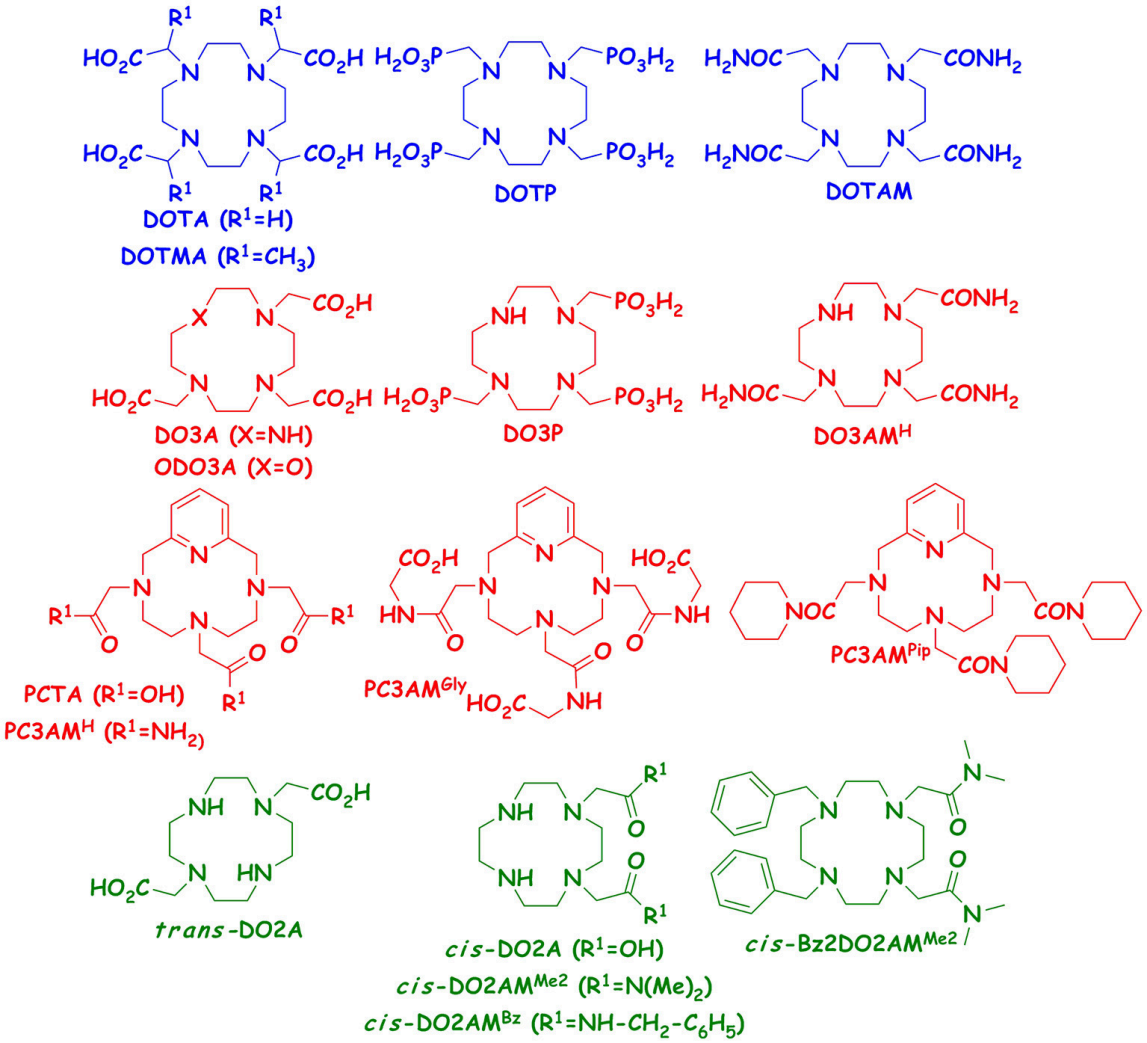
Structures of hexa-, hepta- and octadente ligands studied and compared in this work.

Since we have access to several 12-membered macrocyclic heptadentate ligands (DO3A, ODO3A, PCTA, DO3P, DO3AM, PC3AM^H^, PC3AM^Gly^ PC3AM^Pip^) from our previous studies performed with their Ln(III) complexes, we decided to study the stability and dissociation kinetic properties of the Mn(II) complexes formed with these chelators. We expected to gain more information on how *the rigidity* (DO3A vs. PCTA, or DO3AM^H^ vs. PC3AM^H^), *the nature of the donor atoms* in the macrocycle (DO3A vs. ODO3A) and *the nature of the pendant arms* (DO3A vs. DO3P, i.e., replacement of acetate pendants by phosphonates), DO3A vs. DO3AM (replacement of acetate pendants by amides), or PCTA vs. PCTAM (replacement of acetate pendants by amides for rigidified macrocycle) *and the nature of amide pendants* (PC3AM^H^ vs. PC3AM^Gly^ or PC3AM^Pip^ (replacement of primary amides by secondary and tertiary amides, respectively) affect the physicochemical properties of Mn(II) complexes. The goal of this project was to better understand how these (functional) modifications in the ligand structure affect equilibrium and kinetic behaviors of the corresponding Mn(II) complexes. Such data can help us to design ligands for improved Mn(II) complexation. It should be kept in mind, however, that some of these Mn(II) complexes would not be very efficient relaxation agents because of the lack of inner sphere water molecule. The formulae of the studied ligands are shown in Figure 1.

The stability constants of the Mn(II) complexes were determined by pH-potentiometry and/or ^1^H relaxometry (measuring *T*_1_ relaxation times and plotting 1/*T*_1_ values normalized to 1 mM paramagnetic substance as a function of pH). The kinetic inertness of the Mn(II) complexes have been evaluated by studying the acid catalyzed dissociation or metal/ligand exchange reactions occurring with Cu^2+^ (for [Mn(DOTAM)]^2+^, [Mn(DO3AM^H^)]^2+^, [Mn(DO3A)]^−^, [Mn(PCTA)]^−^ and [Mn(ODO3A)]^−^ complexes), or transCDTA (as in the case of [Mn(DO3A)]^−^ chelate), or DTPA (as in the case of [Mn(DOTP)]^6−^ and [Mn(DO3P)]^4−^ chelates). Based on these data the half-lives were calculated at pH = 1 and 7.4 and compared to those of DO2A and DOTA derivatives published in the literature. The relaxivity values of some complexes were also determined in order to confirm that the relaxivity of the Mn(II) complexes formed with 12-membered heptadentate macrocyclic chelators is purely of outer-sphere in origin since the Mn(II) complexes of macrocyclic ligands with seven or more donor atoms are not expected to have an inner-sphere water molecule (Rocklage et al., 1989).

## Materials and methods

### Materials

The chemicals used in the experiments were of the highest analytical grade. The concentration of the MnCl_2_, CuCl_2_ and ZnCl_2_ solutions was determined by using standardized Na_2_H_2_EDTA solution and eriochrome black T (Mn(II)), murexide (Cu(II) and xylenolorange (Zn(II)) as indicator. The ligands were either prepared by following literature procedures (DOTAM, DO3A, DO3P, PCTA, ODO3A, PC3AM^Gly^) (Amorim et al., 1988; Maumela et al., 1995; Aime et al., 1997; Siaugue et al., 2000; Sun et al., 2003; Rojas-Quijano et al., 2009; Nithyakumar and Alexander, 2016) or obtained from commercial sources (DO3AM^H^ - CheMatech, Dijon (France)). The PC3AM^H^ and PC3AM^Pip^ ligands were prepared by alkylating the pyclen macrocycle prepared according to literature description (Stetter et al., 1981) with a suitable bromoacetamide derivative (see the Supplementary Material for the detailed synthesis and analytical data) available from commercial sources or prepared by following literature synthesis (Kaupang and Bonge-Hansen, 2013).

### Equilibrium measurements

The concentration of the ligand solutions was determined by pH-potentiometric titration in the presence and absence of an excess (5–10 fold) of MnCl_2_. For determining the protonation constants of the investigated ligands, pH-potentiometric titrations were made by means of 0.2 M standardized NaOH in 2 and 3 mM ligand solutions in the pH-range of 1.75–11.85. All equilibrium measurements were performed at a constant 0.15 M NaCl ionic strength at 25 °C. The protonation and the stability constants of the Mn(II) complexes were determined by pH-potentiometric titrations (DOTP, DO3A, PCTA, ODO3A, DO3P) while owing to the slow formation rates of Mn(II) complexes formed with amide type ligands, the stability constants of DOTAM, DOTMA, DO3AM^H^, PC3AM^H^, PC3AM^Gly^, PC3AM^Pip^ complexes were determined by out-of-cell pH-potentiomertry in combination with ^1^H relaxometry. The metal-to-ligand concentration ratio in the solutions was 1:1. For the calculation of protonation constants of the ligands and the log *K*_MnL_ and log *K*_MnLHi_ values of the complexes, mL–pH data pairs (50–180), obtained in the pH range of 1.75–11.85 or *r*_1obs_-pH data pairs (10–15 batch samples) obtained in the pH range of 2–4.5 were used (the samples were equilibrated for 4–7 days). The equilibrium constants characterizing the deprotonation that occurs at basic pH were determined from the data obtained via direct pH-potentiometric titrations performed on the pre-formed complexes.

The pH-potentiometric titrations were carried out with a Methrohm 888 Titrando titration workstation using a Metrohm-6.0233.100 combined electrode. The samples (6.00 mL) were thermostated at 25 °C. The samples were stirred and kept under inert gas (N_2_) to avoid the effect of CO_2_. KH-phthalate (pH = 4.005) and borax (pH = 9.177) buffers were used for the pH-calibration. The method proposed by *Irving et al*. was used for the calculation of H^+^ concentrations from the measured pH values (Irving et al., 1967). A 0.01 M HCl (I = 0.15 M set with NaCl) solution was titrated with standardized NaOH solution of known concentration (approx. 0.2 M). The correction factor obtained as difference of calculated pH and measured pH values was used to calculate the [H^+^] in the samples. The ionic product of water was determined from the same titrations (HCl/NaOH) from the data collected in the pH range of 11.20–11.85.

The relaxometric data were collected using a Bruker Minispec MQ20 NMR relaxometer. The batch samples were equilibrated for 4–7 days (until no further change in the relaxivity was observed for the samples prepared in duplicates) and the *T*_1_ values were measured multiple times. A datapoint plotted on figures represents an average of 5–6 *T*_1_ measurements (Figures S3–S9 in Supplementary Material). The data were fitted by using the molar *r*_1p_ relaxivity value of the MnCl_2_ (7.92 M^−1^s^−1^ at 0.49 T and 25 °C). The equilibrium constants were calculated using the PSEQUAD program (Zékány and Nagypál, 1985).

### Kinetic measurements

The dissociation reactions were followed by UV-vis spectrophotometry at 269 nm for the pyclen derivatives or at 300 nm for other complexes in the pH range of 3.6–5.0 for the exchange reactions with Cu(II) and up to pH = 6.0 when Zn(II) was used as a ligand scavenger. The acid catalyzed dissociation reactions were performed in the HCl concentration range of 0.05–1.0 M. The concentration of the complexes in the samples was 0.25 mM, while the concentration of the exchanging metal ion in the metal initiated transchelation reactions was 10–40 times higher to ensure pseudo-first-order conditions. Relaxometry was used to follow the transchelation reactions of [Mn(DOTAM)]^2+^, [Mn(DO3AM^H^)]^2+^, [Mn(DO3A)]^−^, [Mn(PCTA)]^−^ and [Mn(ODO3A)]^−^ with Cu(II)/Zn(II) ions in the pH range of 3.6–5.5 (Figures S17–S19 in Supplementary Material). The concentration of the complexes in these samples was set to 1.0–2.0 mM to achieve relatively large change during the dissociation reaction. The relaxivity of the complexes is in the range of 1.00–1.44 mM^−1^s^−1^, which increases to 7.92 mM^−1^s^−1^ upon Zn(II) mediated decomplexation. The temperature was maintained at 25 °C and the ionic strength of the solutions was kept constant at 0.15 M with NaCl. *N,N*′-dimethyl- (DMP) and *N*-methyl-piperazine (NMP) buffers (log $K_{2}^{\text{H}}$ = 4.18 and 4.90 at 25 °C and *I* = 0.15 M NaCl, respectively) were used at 0.05 M concentration to keep the pH constant. The pseudo-first-order rate constants (*k*_obs_) were calculated by fitting the absorbance or relaxivity-time data pairs to Equation (1)

(1) $$\begin{array}{r} X_{\text{t}}=\left(X_{0}-X_{\text{e}}\right)e^{-k_{\text{obs}}t}+X_{\text{e}} \end{array}$$

where *X*_t_, *X*_0_, and *X*_*e*_ are the absorbance or relaxivity at time *t*, at the start and at equilibrium, respectively. The calculations were performed with the computer program Micromath Scientist, version 2.0 (Salt Lake City, UT, USA) using a standard least-squares procedure.

### Relaxivity measurements

The longitudinal water proton relaxation rates (*r*_1_ = 1/*T*_1_-1/*T*_w_) were measured at 20 MHz with a Bruker Minispec MQ-20 relaxometer (Bruker Biospin, Germany). Samples were thermostated by using a circulating water bath at 25.0 ± 0.2 °C. The longitudinal relaxation times (*T*_1_) were measured by the inversion-recovery method (180° − τ − 90°), averaging 5–6 data points collected for each concentration point obtained from 14 different τ values (τ values ranging between 0 to at least 6 times the expected *T*_1_). The relaxivity of the complexes was determined by titrating a nearly 1.0 mM ligand solution with a Mn(II) stock solution at pH = 7.22–7.43 (50 mM HEPES buffer, *I* = 0.15 M NaCl, 25 °C). Under these conditions, the only Mn(II) ion containing species present in solution is the [Mn(L)], which was supported by the *r*_1_ vs. pH profiles. The relaxivity of the complex was determined as the slope of the strait line obtained by plotting 1/*T*_1p_ values as a function of Mn(II) concentration for samples with [Ligand] > [Mn(II)]. The relaxivity of [Mn(DOTP)]^6−^ and [Mn(DO3P)]^4−^ was determined in the pH range of 10.0–11.0 and 8.5–9.3, respectively where mainly the [Mn(DO3P)]^4−^ and [Mn(DOTP)]^6−^ complex exists in solution (Figures S4 and S9 in Supplementary Material).

## Results and discussion

### Stability of the Mn(II) complexes

The protonation and complexation equilibria of the macrocyclic ligands and their Mn(II) complexes have been studied by pH-potentiometric and relaxometric methods in the presence of 0.15 M NaCl whose ionic strength mimics the physiological conditions. The protonation constants of the ligands were evaluated by fitting the pH-V(mL) base data pairs collected in the pH-potentiometric titrations. The protonation equilibria of the ligands can be described by Equation (2). The log *K*^H^ values are listed in Table 1. (a comparison of previously published log *K*^H^ data available in the Supplementary Material) together with the constants of H_4_DOTA, *cis-* and *trans-*H_2_DO2A derivatives given for comparison.

(2) $$\begin{array}{r} K_{\text{i}}^{\text{H}}=\frac{\left[{\text{H}}_{\text{i}}\text{L}\right]}{\left[{\text{H}}_{\text{i}-1}\text{L}\right]\left[{\text{H}}^{+}\right]},\text{i}=1,2,\ldots \end{array}$$

As it has been stated before in several publications (Desreux et al., 1981), the first two protonations of these tetraazacyclododecane derivatives occur at the opposite ring nitrogen donor atoms followed by further protonation steps depending on the nature of donor groups incorporated into the pendant arms. The protonation sequence of PCTA and ODO3A and their derivatives differ slightly from that of DOTA and its derivatives owing to the asymmetric nature of the macrocyclic ligands (Aime et al., 1997). The first protonation in these ligands occurs at the nitrogen atom situated *trans* to the pyridine N or etheric O atom. The protonation of a *cis* nitrogen atom in the second step forces the first proton to shift to the other *cis* nitrogen of the macrocycle leaving the *trans* N atom unprotonated (Aime et al., 1997). The substitution of amides for the carboxylate groups (DO3AM^H^, PC3AM^H^, etc.) results in a significant decrease, while the introduction of phosphonate groups (e.g., DO3P) causes a notable increase in the total basicity of the ligands. Not only the value but also the number of the protonation constants is affected by these structural alterations. As it is seen from the data shown in Table 1, the sum of the first two protonation constants (Σ log $\beta_{2}^{\text{H}}$ = log $K_{1}^{\text{H}}+$log $K_{2}^{\text{H}}$) is significantly higher for the acetate derivatives than that for the amide derivative ligands because the number of the negatively charged sidearms decreases, which results in a weaker hydrogen bonding existing between the protonated donor atoms of the macrocycle and the amide group. On the other hand, the basicity of the macrocyclic nitrogen atoms in DO3P is higher than that in DO3A resulting in a noticeably higher ligand basicity. The ligand ODO3A, derived from DO3A by substituting an etheric O-atom for the macrocyclic NH, also displays a drop in the Σ log β${}_{2}^{\text{H}}$ value due to a change in the protonation sequence (Amorim et al., 1988).

**Table 1:** Protonation constants of the hexa-, hepta- and octadentate ligands (*I* = 0.15 M NaCl, 25 °C).

	**log $K_{1}^{\text{H}}$**	**log $K_{2}^{\text{H}}$**	**log $K_{3}^{\text{H}}$**	**log $K_{4}^{\text{H}}$**	**log $K_{5}^{\text{H}}$**	**log $K_{6}^{\text{H}}$**	**Σ log$K_{2}^{\text{H}}$**
DO3A	10.07 (5)	8.93 (6)	4.43 (9)	4.11 (7)	1.88 (7)	–	19.00
DO3AM^H^	9.40 (5)	6.28 (8)	–	–	–	–	15.68
DO3P	12.55 (2)	11.37 (1)	8.57 (2)	7.02(2)	5.36 (2)	1.84 (2)	23.92
ODO3A	8.74 (2)	7.58 (2)	3.99 (3)	2.39(3)	–	–	16.32
PCTA	9.97 (3)	6.73 (5)	3.22 (6)	1.40 (9)	–	–	16.70
PC3AM^H^	8.76 (3)	4.10 (4)	–	–	–	–	12.86
PC3AM^Gly^	8.85 (1)	4.55 (1)	3.81 (1)	3.21 (1)	2.80 (1)	1.38 (1)	13.40
PC3AM^Pip^	8.74 (1)	5.77 (2)	1.42 (9)	–	–	–	14.51
DOTA^[c]^	11.41	9.83	4.38	4.63	1.92	1.58	21.24
DOTMA^[b]^	11.72	9.06	4.74	5.59	1.92	–	20.78
DOTAM	7.31 (1)	6.07 (1)	–	–	–	–	13.38
DOTP	13.6 (2)^[a]^	12.23 (3)	8.63 (5)	7.45 (4)	5.84 (5)	5.02 (5)	25.83
*trans*-DO2A^[d]^	11.69	9.75	3.97	2.68	–	–	21.44
*cis*-DO2A^[d]^	11.44	9.51	4.14	1.55	–	–	20.95
*cis*-DO2AM^Me2, [e]^	10.14	8.38	–	–	–	–	18.52
*cis*-Bz2DO2AM^Me2, [f]^	11.11	8.22	–	–	–	–	19.33
*cis*-DO2AM^Bz2, [f]^	9.62	6.90	–	–	–	–	16.52

[a] 1H- and ^31^P-NMR;

[b] I = 0.1 M KCl (Aime et al., 2011);

[c] 0.1 M KCl (Takács et al., 2014);

[d] Garda et al. (2016);

[e] I = 0.1 M KCl (Forgács et al., 2016);

[f] *I = 0.1 M KCl (Forgács et al., 2017)*.

In order to gain information on the thermodynamic stability of the Mn(II) complexes, samples containing the metal and ligand in 1 to 1 molar ratio were studied by pH potentiometry or ^1^H-relaxometry and in some cases by a combination of the two methods. The titration data were fitted by assuming the existence of [M(L)] and some protonated species ([M(H_i_L)]) at lower pH values. In some cases to fit the data obtained at higher pH, the formation of ternary hydroxido complexes ([M(L)(OH)]) were also included in the equilibrium model. The stability constants (*K*_ML_) and the various protonation forms (*K*_MHiL_) of ML metal chelates are defined by Equations (3)–(5). However, a comparison of the stabilities of different complexes solely on the basic of their stability constants might be misleading because of the differences in ligand basicities. Therefore, we have calculated the pMn values at pH 7.4 defined as pMn = –log [Mn^2+^]_free_ with c_Mn_ = c_L_ = 1 × 10^−5^ M as suggested by Tóth and co-workers (Drahos et al., 2010). The pMn value accounts for the effect of proton competition (ligand basicity and complex protonation) on the stability constant essentially offering the same information as the conditional stability constant. Higher pMn values indicate a higher chelate stability at specified conditions. Considering the above mentioned conditions (c_Mn_ = c_L_ = 1 × 10^−5^ M, pH = 7.4), the minimum value of the pMn is 5, which corresponds to 0% complexation.

(3) $$\begin{array}{lcl} K_{\text{ML}} & = & \frac{\left[\text{M}\left(\text{L}\right)\right]}{\left[\text{M}\right]\left[\text{L}\right]} \end{array}$$

(4) $$\begin{array}{lcl} K_{\text{M}{\text{H}}_{\text{i}}\text{L}} & = & \frac{\left[\text{M}\left({\text{H}}_{\text{i}}\text{L}\right)\right]}{\left[\text{M}\left({\text{H}}_{\text{i}-1}\text{L}\right)\right]\left[{\text{H}}^{+}\right]},\text{ where }i=1,2,\ldots \end{array}$$

(5) $$\begin{array}{lcl} K_{\text{MLOH}} & = & \frac{\left[\text{M}\left(\text{L}\right)\right]}{\left[\text{M}\left(\text{L}\right)\left(\text{OH}\right)\right]\left[{\text{H}}^{+}\right]} \end{array}$$

The ligand DOTA is generally considered as the “gold standard” for metal based systems. The [Mn(DOTA)]^2−^ complex has a pMn value of 9.02 (c_Mn_ = c_L_ = 1 × 10^−5^ M at pH = 7.4) corresponding to log *K*_MnL_ of 19.44. As seen from the data shown in Table 2, the stability constants obtained for the complexes with phosphonate pendant arms ([Mn(DOTP)]^6−^ and [Mn(DO3P)]^4−^) are close to that of [Mn(DOTA)]^2−^; they are the highest among the complexes we studied. The pMn values however, indicate that the conditional stability of these complexes is the lowest among the studied systems. By analyzing the data shown in Table 2 one can conclude that among the studied heptadentate ligands the Mn(II) complex formed with the rigid pyridine macrocyclic PCTA ligand has the highest conditional stability near to physiological conditions. It is higher than that of the [Mn(DO3A)]^−^ complex, although the number and nature of the donor atoms coordinating the Mn(II) ion are nearly the same (4N and 3O) in these ligands. The pMn value characterizing [Mn(PCTA)]^−^ is higher than that calculated for [Mn(DOTA)]^2−^ by 0.7 pM unit while the pMn values of the complexes formed with DO3A, ODO3A and amide derivatives of the rigidified pyclen macrocycles PC3AM^Gly^ and PC3AM^Pip^ ligands are slightly lower than that of [Mn(DOTA)]^2−^ (with a perceptible increase from primary to secondary and to tertiary amide sidearms). These differences can be explained in terms of ligand protonation constants, as it is stated above. The first two protonation constants (log $K_{1}^{\text{H}}$ and log $K_{2}^{\text{H}}$) of DOTA and its derivatives (except for the DOTAM and DO3AM^H^ chelators) are greater than pH 7.4 meaning that the ligands are diprotonated above this pH value, which used in pMn calculations. In contrast, for PCTA and its derivatives only the log $K_{1}^{\text{H}}$ value is higher than 7.4. In case of ODO3A the log $K_{2}^{\text{H}}$ is slightly higher than 7.4 (Table 1) whereas for the DOTA and its derivatives the second protonation occurs above pH = 9.0 (25 °C and *I* = 0.15 M NaCl). Consequently, these ligands exist in their diprotonated form near pH = 7.4 and so the proton competition is more significant in these systems.

**Table 2:** Stability and protonation constants as well as pMn values for the Mn(II) complexes (*I* = 0.15 M NaCl, 25 °C).

	**log *K*_ML_**	**log $K_{\text{ML}}^{\text{H}}$**	**log $K_{\text{MHL}}^{\text{H}}$**	**log $K_{\text{MH}2\text{L}}^{\text{H}}$**	**log $K_{\text{ML}}^{\text{OH}}$**	**pMn^[h]^**
DO3A	16.55 (4)	4.26 (2)	–	–	–	8.66
DO3AM^H^	11.69 (2)	–	–	–	–	7.32
DO3P	17.45 (2)	8.06 (2)	7.05 (1)	5.31 (2)	–	6.43
ODO3A	13.88 (1)	2.77 (5)	–	–	–	8.57
PCTA	16.83 (1) 16.64 (7)^[a]^	1.96(1)	–	–	–	9.74
PC3AM^H^	11.94(3) 11.78 (8)^[a]^	–	–	–	10.90 (4)	7.77
PC3AM^Gly^	13.20 (1)	3.98 (1)	3.14 (1)	2.87 (1)	10.67 (2)	8.37
PC3AM^Pip^	14.05 (2)	–	–	–	11.63 (2)	8.86
DOTA^[b]^	19.44	3.96	–	–	–	9.02
DOTMA^[c]^	18.37^[a]^	4.56^[a]^	–	–	–	8.69
DOTAM	11.96 (1)	–	–	–	–	8.60
DOTP^[d]^	18.98 (1)	8.09 (1)	7.79 (1)	6.74 (1)	–	6.43
*trans*-DO2A^[e]^	14.64	4.40	–	–	–	6.52
*cis*-DO2A^[e]^	15.68	4.15	–	–	–	7.27
*cis*-DO2AM^Me2, [f]^	12.64	–	–	–	–	6.98
*cis*-Bz2DO2AM^Me2, [f]^	11.54	–	–	–	10.44	6.00
*cis*-DO2AM^Bz2, [g]^	10.72	–	–	–	9.44	6.69

[a] 1H-relaxometry;

[b] I = 0.1 M KCl (Takács et al., 2014);

[c] Tircsó and Woods (in preparation);

[d] the 4^th^ protonation constant of the complex is (log K${}_{\text{MH}3\text{L}}^{\text{H}}$ = 5.02 (1);]

[e] Garda et al. (2016);

[f] I = 0.1 M KCl (Forgács et al., 2016);

[g] I = 0.1 M KCl (Forgács et al., 2017);

[h] *The pMn values were calculated at pH = 7.4, c_M_ = c_L_ = 10^−5^ M by using the conditions as suggested by Drahos et al. (2010)*.

**Figure 2:**
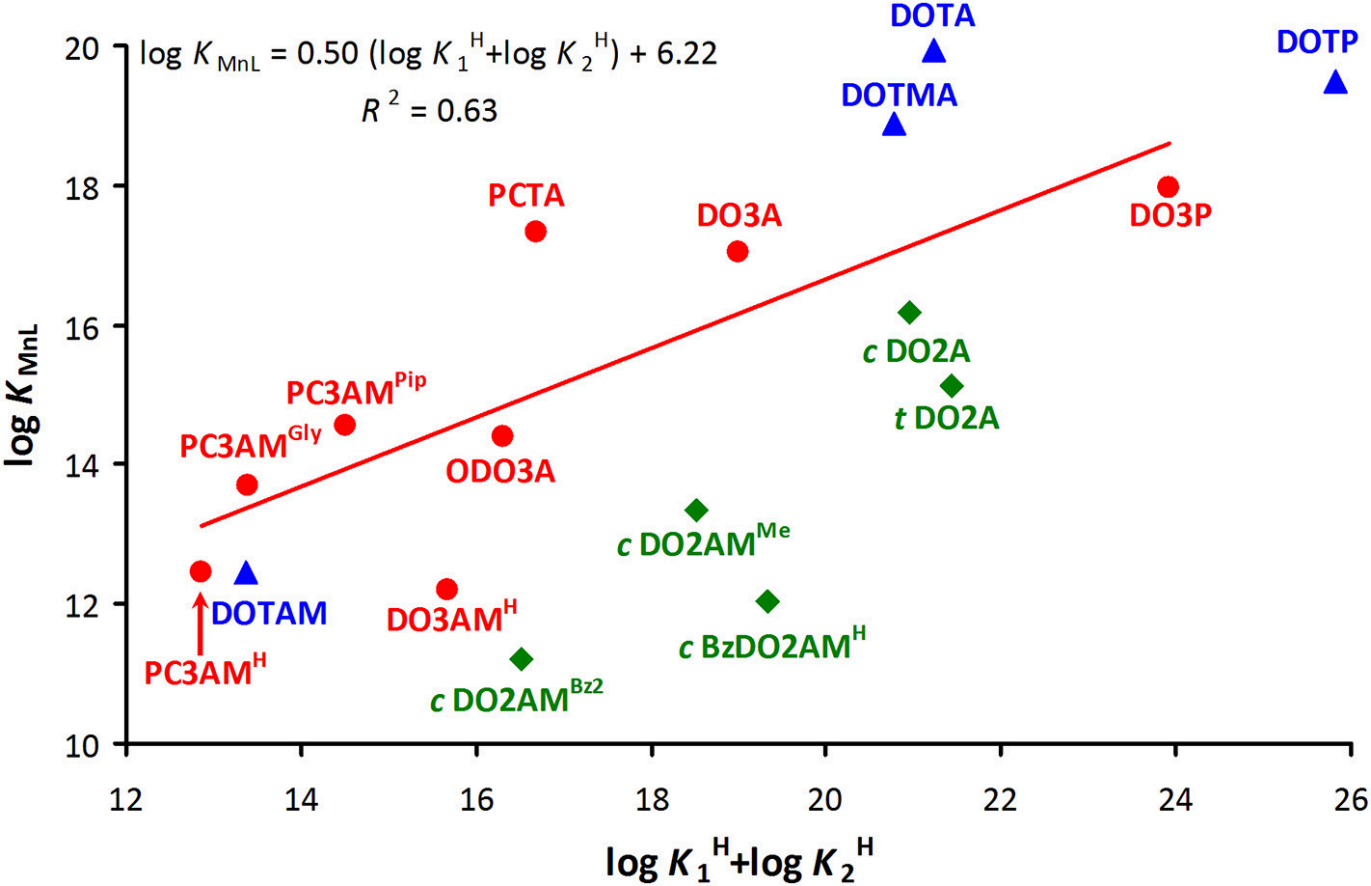
Plot of stability constants (*I* = 0.15 M NaCl and 25 °C) of Mn(II) complexes of 12-membered macrocyclic ligands vs. basicity of the macrocyclic nitrogen atoms (log $K_{1}^{\text{H}}$ + log $K_{2}^{\text{H}}$) of the ligands (green: disubstituted, red: trisubstituted and blue: tetrasubstituted derivatives).

Linear relationships between experimentally measured log *K*_M(L)_ values and ligand basicities were reported for more than 60 years ago (Martell and Calvin, 1952). Later Choppin proposed the inclusion of linear polyamino polycarboxylate systems and demonstrated a single linear correlation between log *K*_M(L)_ values and Σ log $K_{\text{i}}^{\text{H}}$ for polydentate ligands that form five-membered chelate rings with Ln(III) cations (Choppin, 1985). More recently, such empirical relationships have been found for macrocyclic polyamino polycarboxylates as well (Kumar et al., 1995; Huskens et al., 1997). As the diversity of ligands has grown over the years, it has become apparent that a major uncertainty in establishing such relationships is the number of ligand protonation steps that should be included in the calculation of Σ log $K_{\text{i}}^{\text{H}}$ (the basicity of amides, phosphinates, carboxylates and phosphonates differ considerably i.e., these correlations are expected to exist for structurally similar ligands). A similar empirical relationship between the stability (log *K*_[Cu(L)]_) and the sum of the first two log $K_{\text{i}}^{\text{H}}$ values corresponding to the protonation of N-atoms has been shown to hold for Cu(II) complexes of several linear and macrocyclic ligands (Lukes et al., 2001). Even though this relationship ignores the basicity of the side-chain coordinating groups, an analogous approach for Gd(III) complexes with macrocyclic ligands (including 1,4,7-triazacyclonanane, 1,4,7,10-tetraazacyclododecane and 1,4,8,11-tetraazacyclotetradecane with carboxamide, carboxylate and methylenephosphonate pendant arms) gives an acceptable linear correlation with *R*^2^ = 0.86 (log *K*_Gd(L)_ = 1.5 (log $K_{1}^{\text{H}}$ + log $K_{2}^{\text{H}}$) − 9.7) (Brücher et al., 2013). We applied a similar approach to the Mn(II) complexes studied in the current work (Figure 2) This resulted in an unacceptable correlation (*R*^2^ = 0.63) (Figure 2, red points: log *K*_MnL_ = 0.50 (log $K_{1}^{\text{H}}$ + log $K_{2}^{\text{H}}$) + 6.22). However, omission of the data corresponding to PCTA (positive deviation) and DO3AM^H^ (negative deviation) considerably improved the correlation (log *K*_MnL_ = 0.47 (log $K_{1}^{\text{H}}$ + log $K_{2}^{\text{H}}$) + 6.68 with *R*^2^ = 0.89) while the numbers describing the correlation did not change significantly. Figure 2 shows how the weakness of one structural motif can be compensated in part by the strength of another when the effects of various structural features are combined. [Mn(PCTA)]^−^ has higher, whereas [Mn(DO3AM^H^)]^2+^ has lower stability than expected based on the ligand basicity. Likewise, the Mn(II) complex of PCTA-tris(amide), (PC3AM^H^) is less stable than expected while [Mn(PC3AM^Gly^)]^−^ and [Mn(PC3AM^Pip^)]^2+^ have stabilities higher than expected. Our data also indicate that Mn(II) complexes of all the bisamides of *cis*-DO2A reported in the literature have lower stability than expected on the basis of ligand basicity, highlighting the importance of further ligand design.

### Demetallation of the Mn(II) chealtes

**Scheme 1:**
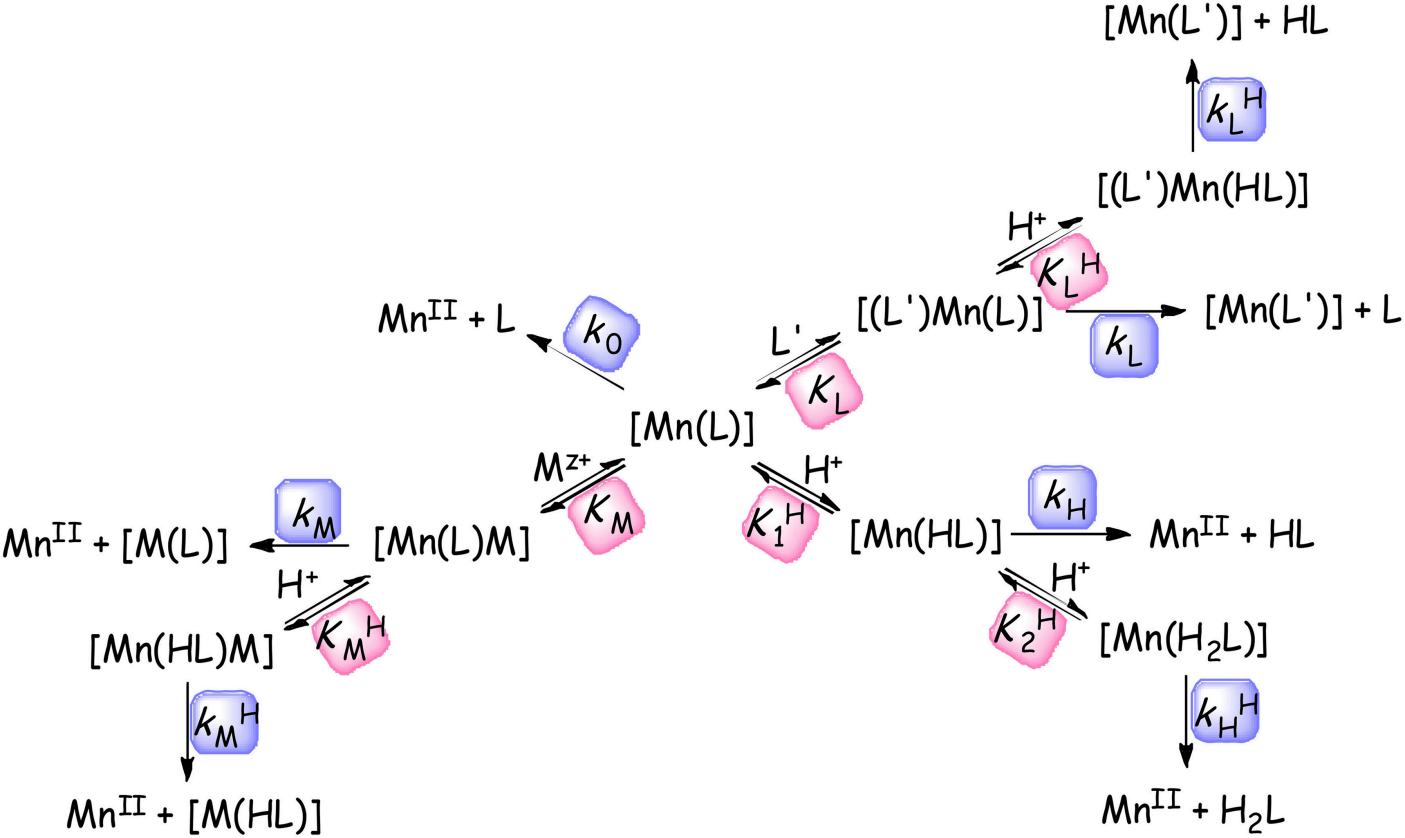
Assumed reaction pathways for the dissociation of Mn(II) complexes in the presence of metal ions and ligands available for exchange reactions (M = Cu(II) or Zn(II), L' = *trans*-CDTA or DTPA, the charges are omitted for clarity).

The kinetic inertness of the Mn(II) complexes is the other important parameter for the complexes considered for use *in vivo*. The inertness of Mn(II) complexes formed with trisubstituted 12-memberd macrocyclic ligands were characterized either by studying the rates of acid catalyzed dissociation or by studying transmetallation reactions occurring between the complexes and a suitable exchanging metal ion such as Zn(II) or Cu(II). The demetallation reactions of labile complexes were performed at high pH by following the ligand exchange reactions with *trans*-CDTA and DTPA ligands. The demetallation reactions were studied in the presence of a large Cu(II)/Zn(II)/*trans*-CDTA/DTPA excess or by using high acid concentration in order to ensure pseudo-first-order conditions (Equation 6). Scheme 1 outlines all the possible dissociation pathways for the Mn(II) complexes. Scheme 1 shows that the dissociation may occur through exchange reactions with other M' metal ion or L' ligand by the assistance of protons. The reactions of the released Mn(II) and L ligand with the exchanging L' and M' reactants are assumed to be very rapid.

The rate constants *k*_0_, *k*_H_, $k_{\text{H}}^{\text{H}}$, *k*_M_, $k_{\text{M}}^{\text{H}}$, *k*_L_, and $k_{\text{L}}^{\text{H}}$ characterize the rate of the spontaneous, proton-assisted, metal-assisted and proton-metal-assisted (when the exchanging metal attacks the protonated or the proton attacks the dinuclear complexes) ligand and proton-ligand reaction pathways, respectively. $K_{1}^{\text{H}}$, $K_{2}^{\text{H}}$, $K_{\text{M}}^{\text{H}}$, and $K_{\text{L}}^{\text{H}}$ are the protonation constants of the [Mn(L)], [Mn(HL)], [Mn(L)M], [Mn(L)(L')], and the stability constant of the dinuclear (*K*_M_) [Mn(L)M] and ternary (*K*_L_) [Mn(L)(L')] complexes, respectively.

The rate of dissociation can be expressed by the Equation (6), where *k*_obs_ is the pseudo-first-order rate constant and [Mn(L)]_*t*_ is the total concentration of the species containing [Mn(L)] complex. The [Mn(L)]_*t*_ will differ slightly depending on the experimental conditions (in the presence of a large metal or ligand excess). As [Mn(L)]_*t*_ = [Mn(L)] + [Mn(HL)] + [Mn(H_2_L)] + [Mn(L)M] + [Mn(L)(L')] + [Mn(HL)(L')] for reactions performed at high pH (in the pH range of 3.0–5.0 often applied in Cu(II) exchange reactions) will involve fewer protonated species while the acid catalyzed dissociation reactions run in the strongly acidic media (0.1–1.0 M acid concentration range) predominantly contain protonated species ([Mn(L)]_t_ = [Mn(L)]+[Mn(HL)]+[Mn(H_2_L)]).

(6) $$\begin{array}{r} -\frac{\text{d}{\left[\text{MnL}\right]}_{\text{t}}}{\text{dt}}=k_{\text{obs}}{\left[\text{MnL}\right]}_{t} \end{array}$$

By taking into account the various complex species and the possible reaction pathways as well as the protonation and stability constants of the reactive intermediates, the following equation (Equation 7) can be derived to describe the rates of dissociation.

(7) $$\begin{array}{r} k_{\text{obs}}=\frac{k_{0}+k_{1}\left[{\text{H}}^{+}\right]+k_{2}{\left[{\text{H}}^{+}\right]}^{2}+k_{3}\left[{\text{M}}^{2+}\right]+k_{4}\left[{\text{M}}^{2+}\right]\left[{\text{H}}^{+}\right]+k_{5}\left[{\text{L}}^{\prime }\right]+k_{6}\left[{\text{L}}^{\text{′}}\right]\left[{\text{H}}^{+}\right]}{1+{K_{1}}^{\text{H}}\left[{\text{H}}^{+}\right]+{K_{1}}^{\text{H}}{K_{2}}^{\text{H}}{\left[{\text{H}}^{+}\right]}^{2}+K_{\text{M}}\left[{\text{M}}^{2+}\right]+{K_{\text{M}}}^{\text{H}}\left[{\text{M}}^{2+}\right]\left[{\text{H}}^{+}\right]+K_{{\text{L}}^{\text{′}}}\left[{\text{L}}^{\text{′}}\right]+{K_{{\text{L}}^{\text{′}}}}^{\text{H}}\left[{\text{L}}^{\text{′}}\right]\left[{\text{H}}^{+}\right]} \end{array}$$

where *k*_1_ = *k*_H_·$K_{1}^{\text{H}}$*, k*_2_ = $k_{\text{H}}^{\text{H}}\cdot$$K_{1}^{\text{H}}\cdot$$K_{2}^{\text{H}}$*, k*_3_ = *k*_M_·*K*_M_, *k*_4_ = $k_{\text{M}}^{\text{H}}\cdot$$K_{\text{M}}^{\text{H}}$, *k*_5_ = *k*_L_·*K*${}_{{\text{L}}^{\text{′}}}$ and *k*_6_ = $k_{\text{L}}^{\text{H}}\cdot$$K_{{\text{L}}^{\text{′}}}^{\text{H}}$. By fitting the experimental *k*_obs_ data to Equation (7) resulted in the rate constants characterizing spontaneous (*k*_0_, s^−1^), acid catalyzed (*k*_1_, M^−1^s^−1^ and *k*_2_, M^−2^s^−1^) and metal-assisted (*k*_3_, M^−1^s^−1^) dissociation for the Mn(II) complexes that are listed in Table 3. For the calculation we had to have some information about the importance of different reaction pathways. The role of the metal-proton-assisted (*k*_4_, M^−2^s^−1^), ligand (*k*_5_, M^−1^s^−1^) and ligand-proton-assisted (*k*_6_, M^−2^s^−1^) pathways in these demetallation reactions were negligible and in most cases the protonation and stability constants of the intermediates ($K_{1}^{\text{H}}$, $K_{2}^{\text{H}}$*, K*_M_, and $K_{{\text{L}}^{\text{′}}}^{\text{H}}$) could not be determined because of their very low values.

**Figure 3:**
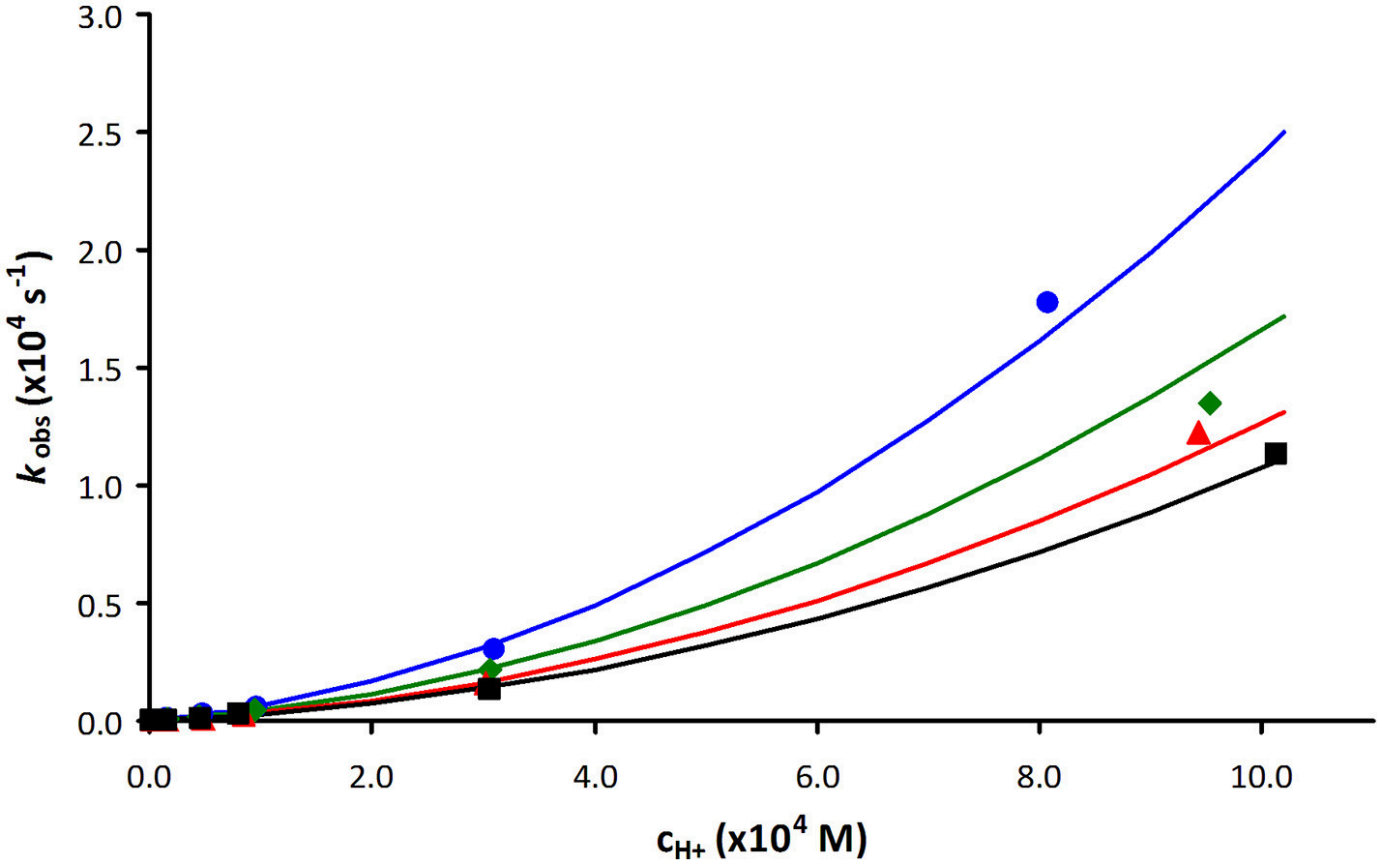
Plot of the pseudo-first-order rate constants (*k*_obs_) as a function of Zn(II) and H^+^ ion concentration for [Mn(PCTA)]^−^ (blue: 10-fold Zn(II) excess, green: 20-fold Zn(II) excess, red: 30-fold Zn(II) excess and black: 40-fold Zn(II) excess).

**Figure 4:**
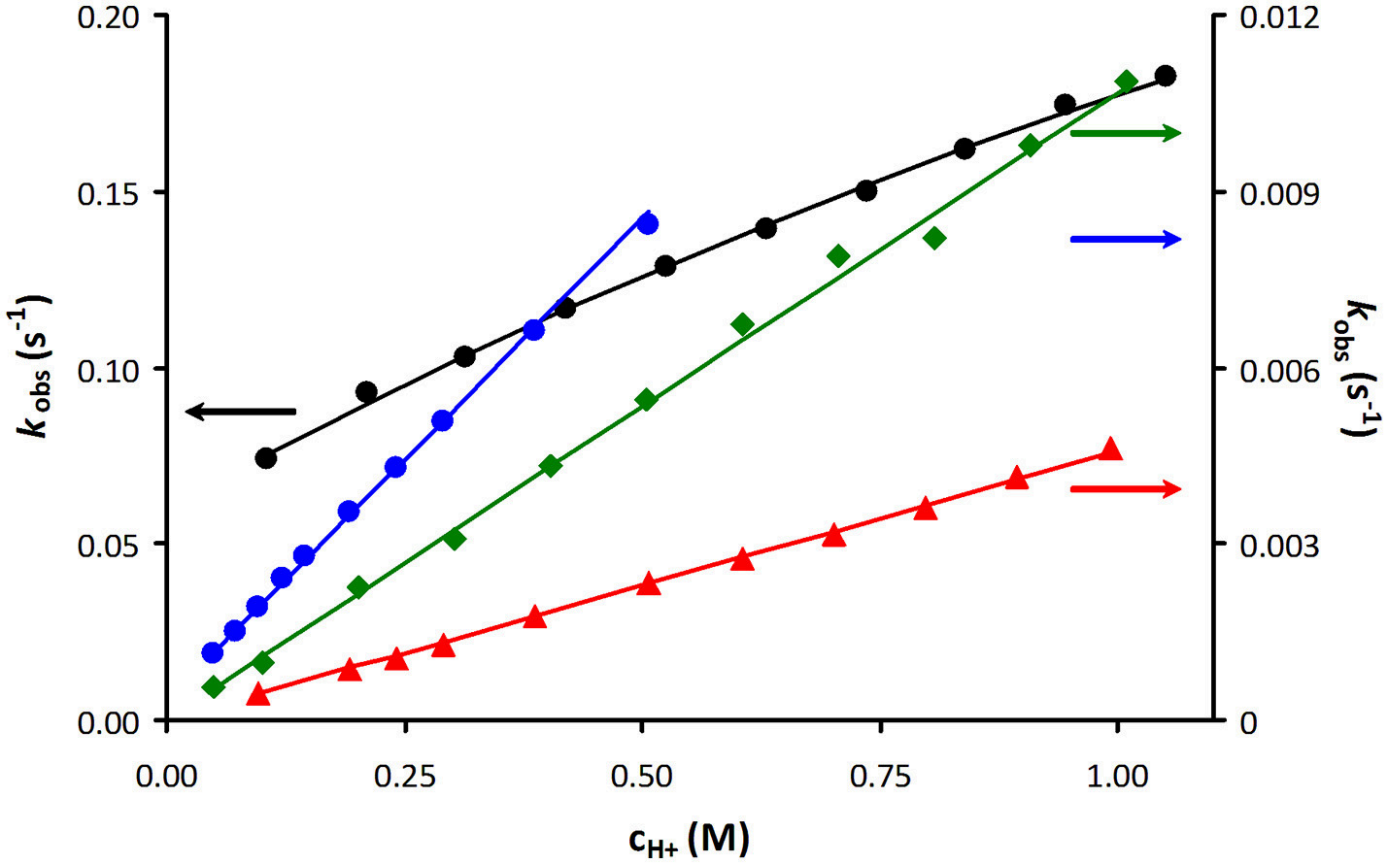
Plot of the pseudo-first-order rate constants (*k*_obs_) as a function of H^+^ ion concentration for [Mn(PCTA)]^−^ (black), [Mn(PC3AM^H^)]^2+^ (green), [Mn(PC3AM^Gly^)]^−^ (blue) and [Mn(PC3AM^Pip^)]^2+^ (red).

As an example, Figure 3 shows a representative plot of *k*_obs_ values as a function of acid concentration obtained for [Mn(PCTA)]^−^ during the Zn(II) induced transmetallation reactions in the pH range of 3.09–5.88 (some more data are included in the ESI). The rates of acid catalyzed dissociation reactions obtained for PCTA and its amide derivatives are shown in Figure 4.

**Table 3:** Rate constants of spontaneous (*k*_0_), proton-assisted (*k*_1_ and *k*_2_), metal-ion-assisted (*k*_3_) pathways and half-lives characterizing the dissociation of Mn(II) complexes (*I* = 0.15 M NaCl, 25 °C).

	***k*_0_ (s^−1^)**	***k*_1_ (M^−1^s^−1^)**	***k*_2_ (M^−2^s^−1^)**	***k*_3_ (M^−1^s^−1^)**	***t*_1/2_ (s) 0.1 M HCl**	***t*_1/2_ (h) pH = 7.4 c_M_ = 10^−5^ M**
[Mn(PCTA)]^−^	(7.0 ± 0.1) × 10^−2[b]^	(8.2 ± 0.7) × 10^−2[a]^ (1.09 ± 0.01) × 10^−1[b]^	(3.5 ± 0.4) × 10^2[a]^	(1.8 ± 0.3) × 10^−6[a]^	8.6	5.9 × 10^4^
[Mn(PC3AM^H^)]^2+^	–	(1.07 ± 0.01) × 10^−2^	–	–	6.5 × 10^2^	4.5 × 10^5^
[Mn(PC3AM^Gly^)]^−^	(3.4 ± 0.2) × 10^−4[c]^	(1.64 ± 0.02) × 10^−2[c]^	–	–	3.5 × 10^2^	^[l]^
[Mn(PC3AM^Pip^)]^2+^	–	(4.64 ± 0.04) × 10^−3^	–	–	1.5 × 10^3^	1.0 × 10^6^
[Mn(DO3A)]^−^	–	0.45 ± 0.03	(3.2 ± 0.3) × 10^2^	–	0.21	1.1 × 10^4^
[Mn(DO3AM^H^)]^2+^	–	0.94 ± 0.02	–	–	7.4	5.2 × 10^3^
[Mn(DO3P)]^4−^^[e]^	–	(2.4 ± 0.2) × 10^5^	(2.2 ± 0.3) × 10^14^	–	^[k]^	3 × 10^−3^
[Mn(ODO3A)]^−^^[d]^	–	27 ± 2	(1.5 ± 0.3) × 10^5^	–	2.8 × 10^−2^	1.8 × 10^2^
[Mn(DOTP)]^6−^^[e]^	–	(2.35 ± 0.29) × 10^5^	(8.35 ± 0.8) × 10^14^	–	^[k]^	1.3 × 10^−3^
[Mn(DOTAM)]^2+^	–	(1.6 ± 0.03) × 10^−2^	–	–	4.3 × 10^2^	3.0 × 10^5^
[Mn(DOTMA)]^2−[f]^	1.04 × 10^−4^	3.96 × 10^−5^	–	–	6.2 × 10^3^	^[l]^
[Mn(DOTA)]^2−[g]^	1.8 × 10^−7^	0.04	1.6 × 10^3^	1.5 × 10^−5^	4.3 × 10^−2^	1.1 × 10^3^
[Mn(*trans*-DO2A)]^[h]^	–	85	3.0 × 10^6^	–	2.3 × 10^−5^	48
[Mn(*cis*-DO2A)]^[h]^	–	100	1.6 × 10^6^	–	4.3 × 10^−5^	58
[Mn(*cis*-DO2AM^Me2^)]^2+[i]^	–	8.7	–	–	0.79	556
[Mn(*cis*-BzDO2AM^H^)]^2+, [j]^	–	36	–	–	0.19	136
[Mn(*cis*-DO2AM^Bz2^)]^2+, [j]^	–	38	–	–	0.18	126

[a] relaxometry, the stability constant of the dinuclear intermediate (K_M_) was found to be 38 ± 6 M^−1^;

[b] stopped-flow in highly acidic condition where the k_0_ is the spontaneous and k_1_ is the proton-assisted dissociation of the [Mn(HL)] complex;

[c] the k_0_ is the spontaneous and k_1_ is the proton-assisted dissociation of the [Mn(H_3_L)] complex;

[d] the K${}_{1}^{\text{H}}$ was fixed to 600 based determined by pH-potentiometry;

[e] ligand exchange reaction with trans-CDTA^4−^ or DTPA^5−^ in the pH range 8-9.5, the stability constant of the protonated intermediate (K${}_{1}^{\text{H}}$) was found to be (2.0 ± 0.4) × 10^8^, the K_H_ was fixed to 10^8.06^;

[f] Tircsó and Woods (in preparation) in 1.0 M KCl, where the k_0_ is the spontaneous and k_1_ is the proton-assisted dissociation of the [Mn(H_2_L)] complex;

[g] the stability constant of the [Mn(DOTA)Zn] dinuclear intermediate (K_M_) was found to be 68 M^−1^ (Drahos et al., 2011);

[h] Garda et al. (2016);

[i] I = 0.1 M KCl (Forgács et al., 2016);

[j] I = 0.1 M KCl (Forgács et al., 2017);

[k] the half-life (t_1/2_) corresponding to 0.1 M acid concentration were not calculated since the dissociation reactions were carried out at high pH (where [Mn(DO3P)]^4−^ and [Mn(DOTP)]^6−^ exist in multiple protonated forms up to pH = 9.0 and their dissociation is extremely fast), i.e., the rate constants were determined only for the fully deprotonated species;

[l] *the half-life (t_1/2_) was not calculated since the dissociation reactions were carried out under acidic conditions where a protonated complex exists*.

The *k*_obs_ values increase with increasing H^+^ ion concentration in all cases (*k*_1_, *k*_2_) and either increase (*k*_3_) or remain unaffected by increasing the exchanging metal ion concentration. It is difficult to directly compare the rate constants that characterize the different reaction pathways. Therefore, the half-lives (*t*_1/2_) of the dissociation reactions of Mn(II) complexes were calculated under physiological conditions (pH = 7.4, 10 μM concentration of the exchanging metal ion) and also for 0.1 M H^+^ concentration since the most inert Mn(II) complexes were studied in acidic milieu (Table 3).

The comparison of the *k*_1_ (rate constant characterizing the acid catalyzed dissociation) or half-live (*t*_1/2_) values shows that the dissociation kinetics of the Mn(II) complexes of 12-membered macrocyclic ligands strongly depends on the rigidity of the macrocycles, the nature of donor atoms present in the macrocycle, as well as on the metal binding moieties attached to the nitrogen atoms of the macrocycles. For a class of very similar ligands (i.e., 12-membered macrocycles), the rate of acid catalyzed dissociation (*k*_1_) was found to differ as much as eight orders of magnitude (2.4 × 10^5^ M^−1^s^−1^ for DO3P vs. 4.64 × 10^−3^ M^−1^s^−1^ for PC3AM^Pip^), which underlines the importance of both the macrocyclic backbone and the metal binding sidearms in ligand design. As seen from the data shown in Table 3, the replacement of the secondary amine (>NH in DO3A) in the macrocycle by etheric oxygen atom (-O- as in ODO3A) resulted in a 50 fold increase in the *k*_1_ value of the Mn(II) complexes, which indicates that the given structural modification should not be considered for Mn(II) chelators. On the other hand, the incorporation of the secondary amine moiety (>NH) into a pyridine ring has a beneficial effect on the kinetic parameters as evidenced by the 5-fold decrease of the *k*_1_ value of its Mn(II) complex. When analyzing the effect of the nature of pendant arms on the dissociation kinetic properties, it can be concluded that the inertness of Mn(II) complexes of amide derivatives is higher than that of Mn(II) complexes formed with ligands having acetate or especially, phosphonate sidearms. This behavior is clearly related to the decreased basicity of the amide based ligands as well as the hindered proton transfer from the protonation site to the ring nitrogen donor atom(s), which is essential for the acid-assisted path. It was previously proved that the substitution of a phosphonate for the carboxylate group can facilitate the proton transfer to the macrocyclic nitrogen since the phosphonate groups (in acidic solutions) can be protonated even when they are metal-bound (Kálmán et al., 2008). The replacement of the acetates by primary amides resulted in noticeable increase in the kinetic inertness going from PCTA to its trisamide derivatives (PC3AMH). Further improvement was observed going from the primary (PC3AM^H^) toward the tertiary amide (PC3AM^Pip^) complexes (the *k*_1_ values decrease from 0.109 M^−1^s^−1^ observed for the anionic PCTA complex to 0.0107 M^−1^s^−1^ and 0.00464 M^−1^s^−1^ for the cationic Mn(II) complexes of PC3AM^H^ and PC3AM^Pip^, respectively). This tendency is analogous to the kinetic behavior of Ln(III) complexes of DOTA-tetraamides (Aime et al., 1999; Pasha et al., 2007). A break in this trend is apparent for the [Mn(PC3AM^Gly^)]^−^ complex, which is due to the presence of charged (and protonatable) groups in this ligand. The sterically more hindered amide [Mn(PC3AM^Pip^)]^2+^ complex displays surprisingly high kinetic inertness as evidenced by its *k*_1_ rate constant, which is significantly lower than that of [Mn(DOTA)]^2−^) in spite of its lower denticity.

**Figure 5:**
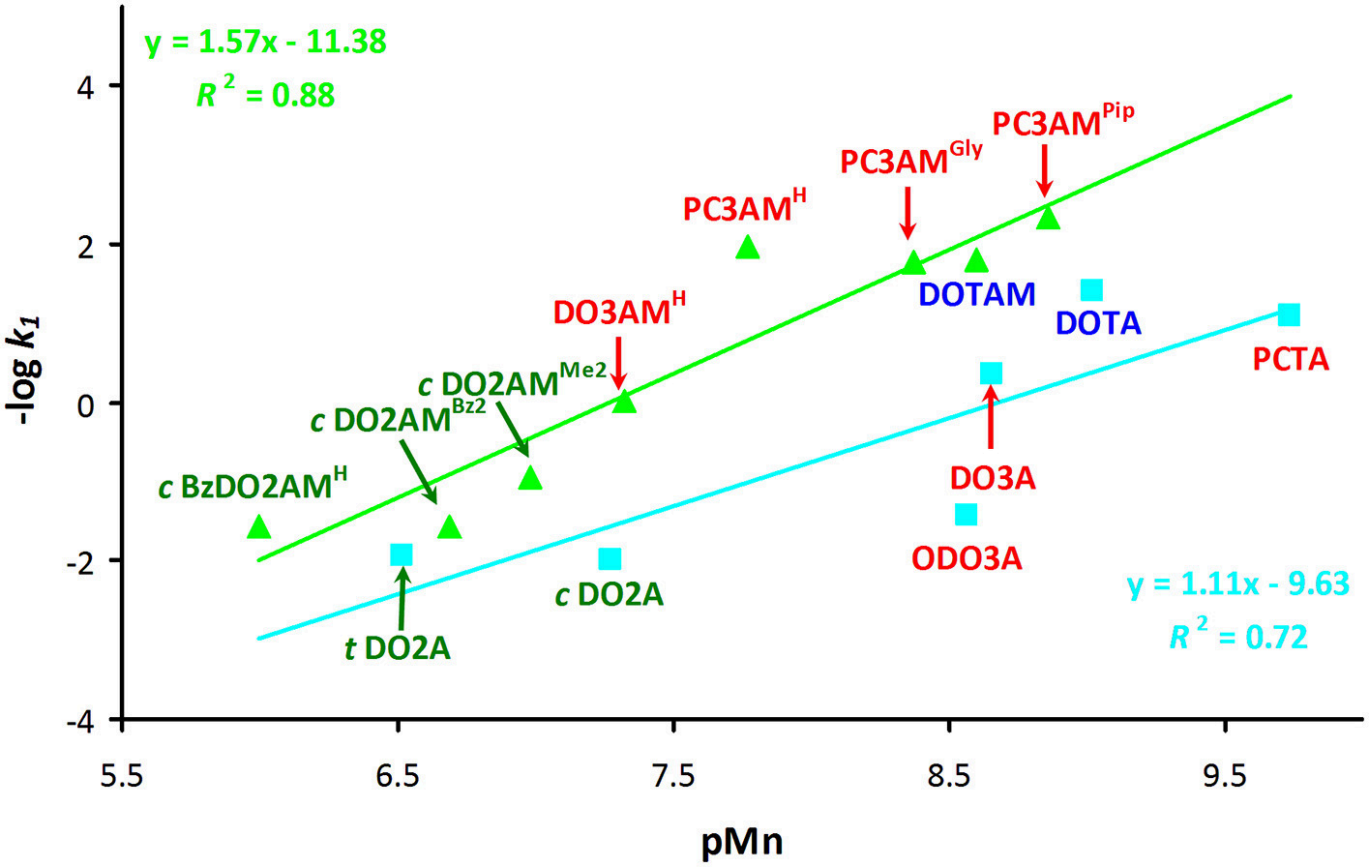
Plot of –log *k*_1_ values (*k*_1_ is the rate constant of the acid-assisted dissociation) as a function of pMn for acetate (light blue) and amide (light green) derivatives of 12-membered macrocycles (*I* = 0.15 M NaCl and 25 °C). Disubstituted ligands are shown in green, trisubstituted derivatives are in red and tetrasubstituted derivatives are in blue.

We have attempted to correlate the dissociation kinetic data and the stability or pMn values of the Mn(II) complexes by plotting the –log *k*_1_ values against the basicity of the nitrogen atoms of the ligands (log $K_{1}^{\text{H}}$, log $K_{2}^{\text{H}}$, and log $K_{1}^{\text{H}}$ + log $K_{2}^{\text{H}}$). Plots of the –log *k*_1_ values against the first (log $K_{1}^{\text{H}}$) and second (log $K_{2}^{\text{H}}$) protonation constants are included in the supporting information (Figures S11–S14). Although no direct correlation is expected to exist between thermodynamic and kinetic parameters, we could observe a linear correlation when the –log *k*_1_ values were plotted as a function of pMn values (Figure 5). However, the acetate (blue line) and amide (green line) systems had to be considered separately, although it should be emphasized that we had very limited data to work with. The basicity of amide ligands weaker than that of the acetate derivatives, thus the Mn(II) complexes of the amide derivatives tend to dissociate more slowly via the proton assisted dissociation path. This is reflected by the fact that the correlation curve for the amide complexes runs above the line of the acetate derivatives and it has slightly larger slope. This observation strongly implies if one could design amide ligands that would form complexes with higher thermodynamic (and conditional) stability, then the kinetic inertness of such complexes is also expected to increase.

### Relaxivity of the Mn(II) complexes

The relaxivity (*r*_1p_, mM^−1^s^−1^) is defined as the longitudinal water proton relaxation rate in a solution containing 1.0 mM concentration of the paramagnetic species. It characterizes the efficiency of a paramagnetic metal chelate to enhance the solvent proton relaxation rate at a given magnetic field (usually 20 MHz) and temperature. The inner-sphere contribution to relaxivity is directly proportional to the number of metal bound water molecules (*q*), and therefore, relaxivity values may provide some useful information on the hydration state of paramagnetic complexes of similar molecular weights and electronic relaxation times. As published in the literature, the Mn(II) complex formed with the heptadentate ligand DO3A does not possess a metal bound water molecule (*q* = 0), and therefore, it has low relaxivity (1.30 mM^−1^s^−1^) originating solely from second and outer sphere contributions (Rolla et al., 2013). The *r*_1p_ values of the Mn(II) complexes formed with heptadentate 12-membered macrocyclic ligands were determined at 0.5 *T* field strength and 25 °C and reported in Table 4 along with some relaxivity data published for the complexes of hexa-, hepta- and octadentate ligands. The relaxivity of the [Mn(PCTA)]^−^ and [Mn(ODO3A)]^−^ complexes are indeed very similar to that of the [Mn(DO3A)]^−^ suggesting the absence of metal bound water molecules in these complexes as well. In fact, the X-ray crystal structure of the Mn(II) chelate formed with tris(ethyl ester) of PCTA confirms the absence of the metal bound solvent molecule in the [Mn(PCTA-OEt_3_)]^2+^ complex (Wen et al., 2012) suggesting that this is also true for the PCTA complex. The Mn(II) complexes of amide derivatives show even lower relaxivity, which can be explained by weaker second and outer sphere contributions (for example, X-ray crystallography has shown that [Mn(DO3AM^H^)]^2+^ does not have an inner sphere water molecule) (Wang and Westmoreland, 2009) whereas increased second and outer sphere effects are likely responsible for the slight increase in the relaxivities observed for [Mn(DOTP)]^6−^ and [Mn(DO3P)]^4−^ in the pH ranges of 10.0–11.0 and 8.5–9.3, respectively, where the deprotonated complex exists in solution. Below these pH ranges the phosphonate moieties undergo protonation, which further increases the relaxivity (2.60–2.90 mM^−1^s^−1^ values were observed near pH = 7.4, see Supplementary Material). Similar increase in the relaxivity (to 3.20 mM^−1^s^−1^ at 20 MHz, 37 °C) is observed for the [Mn(DOTA)]^2−^ complex upon its protonation and formation of the thermodynamically stable diprotonated [Mn(H_2_DOTA)] complex that exists as a non-hydrated chelate both in solution and in the solid state (Wang and Westmoreland, 2009). The relaxivity of the diprotonated species at t → 0 time point was determined from the extrapolation of the relaxivity vs. time data collected after acidification of the samples containing the complexes. The X-ray crystallographic structure of [Mn(H_2_DOTA)] indicates the protonation of two *trans* carboxylate moieties (e.g., the coordination around the metal ion is similar to that observed in [Mn(*trans*-DO2A)] as determined by DFT calculations). Thus, the relaxivity of the diprotonated [Mn(H_2_DOTA)] and [Mn(H_2_DOTMA)] complexes is expected to be similar to that of [Mn(*trans*-DO2A)] (when *q* = 0). The value obtained by us is considerably higher, which highlights the importance of prototropic exchange as a useful tool to improve the relaxivity of the complexes (however, it has to be underlined that the protonation of the complex generally leads to a decrease in kinetic inertness). A similar relaxivity enhancing role of prototropic exchange was demonstrated for Gd(III) complexes of phosphonate derivatives such as ([Gd(DOTP)]^5−^ and [Gd(DOTA-4Amp)]^5−^) (Avecilla et al., 2003; Kálmán et al., 2007).

**Table 4:** Relaxivities (*r*_1p_) determined for selected Mn(II) complexes (at 0.5 *T* field strength, 25 °C and pH = 7.4).

**Chelate**	***r*_1p_ (mM^−1^s^−1^)**	**Chelate**	***r*_1p_ (mM^−1^s^−1^)**
[Mn(PCTA)]^−^	1.50	[Mn(DOTP)]^6−^	2.37^[e]^
[Mn(PC3AM^H^)]^2+^	1.21	[Mn(DOTAM)]^2+^	1.11, 0.96^[f]^
[Mn(PC3AM^Gly^)]^−^	1.44	[Mn(DOTMA)]^2−^/[Mn(H_2_DOTMA)] (*q* = 0)	1.76/2.98^[g]^
[Mn(PC3AM^Pip^)]^2+^	1.28	[Mn(DOTA)]^2−^/[Mn(H_2_DOTA)] (*q* = 0)	1.25/3.20^[f]^
[Mn(DO3A)]^−^	1.18-1.31, 1.30^[a]^	[Mn(*trans*-DO2A)]	1.50^[a]^
[Mn(DO3AM^H^)]^2+^	1.33	[Mn(*cis*-DO2A)]	2.10^[a]^
[Mn(DO3P)]^4−^	2.23^[b]^	[Mn(*cis*-DO2AM^Me2^)]^2+^	2.50^[h]^
[Mn(ODO3A)]^−^	1.40	[Mn(*cis*-BzDO2AM^H^)]^2+^	3.80^[i]^
[Mn(AAZTA)]^2−^, ^[c]^	1.60^[d]^	[Mn(*cis*-DO2AM^Bz2^)]^2+^	3.50^[i]^

[a] Rolla et al. (2013);

[b] determined in the pH range of 8.5–9.3 where mainly the [Mn(DO3P)]^4−^ complex exists in solution;

[c] AAZTA = 6-amino-6-methylperhydro-1,4-diazepine tetraacetic acid;

[d] Tei et al. (2011);

[e] determined in the pH range of 10.0–11.0 where the deprotoanted complex exists in solution;

[f] Wang and Westmoreland (2009) measured at 0.5 T field strength and 37 °C;

[g] Tircsó and Woods (in preparation);

h Forgács et al. (2016);

[i] *Forgács et al. (2017)*.

## Conclusions

In this work, we summarized the results of our studies on the equilibrium and kinetic properties of Mn(II) complexes of several trisubstituted 12-membered macrocyclic ligands tested in our laboratory during the past 5–6 years. Originally, these ligands were intended for Ln(III) complexation. In this project, these structurally extremely diverse ligands were investigated for their Mn(II) chelating ability and we believe that the results presented here will help us to better understand the relationship that exists between the ligand structures and the thermodynamic/kinetic/relaxometric properties of their Mn(II) complexes. The stability constants of the complexes were determined by pH-potentiometry and often supported by ^1^H-relaxometry. As expected, decreasing the denticity and basicity of the parent ligand DOTA (i.e., removing one metal binding pendant arm to afford DO3A) resulted in a drop in the stability of the Mn(II) complex. This decrease can be compensated by incorporating the fourth (secondary) nitrogen atom into a pyridine ring (e.g., PCTA) or replacing the secondary amine with an etheric oxygen atom. The substitution of primary amides for the acetates also resulted in a drop in the stability constant (DO3AM^H^ or PC3AM^H^), but the stability increased as the primary amides (PC3AM^H^) were replaced by secondary (PC3AM^Gly^) or tertiary amide (PC3AM^Pip^) moieties. The ligands incorporating phosphonate pendant arms were found to form the most stable Mn(II) complexes but their conditional stability at pH = 7.4 was the lowest. Very similar conclusion was derived by analyzing the dissociation kinetics data. Among the studied complexes, [Mn(DO3P)]^4−^ was found to be the most labile while the Mn(II) complex of the rigid macrocycle (pyclen) based tertiary amide chelator PC3AM^Pip^ has the highest kinetic inertness whose acid catalyzed dissociation rate is nearly 8 orders of magnitude lower than that of [Mn(DO3P)]^4−^. By considering the structure of PC3AM^Pip^ it can be concluded that the rigidity of the macrocycle is one of the key structural features that determine the dissociation kinetic properties of Mn(II) complexes. Further improvements can be achieved by proper selection of the donor atoms in the metal binding sidearms attached to the nitrogen atoms of the macrocycles. The tertiary amide coordinating sidearm appears to be one of the best candidates in this respect. The Mn(II) complexes of the studied heptadentate ligands are poor *T*_1_ relaxation agents because they do not have a metal bound water molecule. However, the trends observed for Gd(III) complexes seems to hold for the Mn(II) chelates as evidenced by the low relaxivity of the amide based systems or by the increase in the relaxivity due to accelerated prototropic exchange in [Mn(DOTP)]^6−^ and [Mn(DO3P)]^4−^.

## Author contributions

The ligand synthesis was accomplished by ZK and GT. Equilibrium and relaxometric studies were performed by ZG, EM, FKK, RB, ZB, and GT while were analyzed with the help of IT and EB. Kinetic studies were performed by ZG, EM, FKK, and GT and the data were evaluated with the help of IT and EB. The manuscript was written through contributions of all authors. All authors have given approval to the final version of the manuscript.

### Conflict of interest statement

The authors declare that the research was conducted in the absence of any commercial or financial relationships that could be construed as a potential conflict of interest.
