# Supplementary material for: Effect of the Nature of Donor Atoms on the Thermodynamic, Kinetic and Relaxation Properties of Mn(II) Complexes Formed With Some Trisubstituted 12-Membered Macrocyclic Ligands
Source: Front Chem. 2018 Aug 13;6:232. doi: 10.3389/fchem.2018.00232 (PMC6099102; doi:10.3389/fchem.2018.00232)
Supplement: Supplementary file 1 [file Presentation_1.pdf]

Supplementary Material

Effect of the nature of donor atoms on the thermodynamic, kinetic and relaxation properties of Mn(II) complexes formed with some trisubstituted 12-membered macrocyclic ligands

Zoltán Garda,[a] Enikő Molnár,[a] Ferenc K. Kálmán[*a], Richárd Botár [a], Viktória Nagy,[a] Zsolt Baranyai,[a,†] Ernő Brücher,[a] Zoltán Kovács,[b] Imre Tóth,[a] Gyula Tircsó[* a]

- **Correspondence:** Corresponding Author: kalman.ferenc@science.unideb.hu gyula.tircso@science.unideb.hu

[a] Department of Inorganic and Analytical Chemistry, Faculty of Science and Technology, University of Debrecen, H-4010, Debrecen, Egyetem tér 1, Hungary

[b] Advanced Imaging Research Center, The University of Texas Southwestern Medical Center, 5323 Harry Hines Blvd, Dallas, TX 75390, USA

[†] current address of the coauthor is: Bracco Imaging spa, Bracco Research Centre, Via Ribes 5, 10010 Colleretto Giacosa (TO), Italy

1 Supplementary Data

Details of the synthesis:

The ligands $PC3AM^{H}$ and $PC3AM^{Pip}$ were prepared by alkylating the pyclen macrocycle with a suitable bromoacetamide derivative obtained form commercial sources (2-bromoacetamide) or prepared according to published procedures (2-bromo-1-(piperidin-1-yl)ethanone).

$PC3AM^{H}$: A solution of pyclen (0.25 g, 1.21 mmol), commercially available 2-bromoacetamide (0.55 g, 4.00 mmol) and potassium carbonate (1.20 g, 8.68 mmol) in anhydrous acetonitrile was heated to 65 °C with stirring for 3 days. The solution was then filtered and the filtrate evaporated under vacuum. The resulting residue was redissolved water and acetonitrile mixture (50:50 by volume and s purified by preparative HPLC using Luna 10u-Prep C18(2) 100A (250×21.20 mm) column and ACN:H2O/TFA was applied as eluent (the TFA was present only in water at 0.005 M concentration). The product was collected by freeze-drying as white fluffy solid. Yield: 0.33 g (45%).

^{1}H NMR (270 MHz, D_2O): δ 8.06 ppm (t, 1H, aromatic ring), 7.52 ppm (d, 2H, aromatic ring), 4.82 ppm (s, 4H, $ArCH_2Nring$), 4.25 ppm (s, 4H, glycinate methylene), 3.64 ppm (s, 2H, glycinamide methylene), 3.54 ppm (b, 4H, $NringCH_2$), 2.97 ppm (b, 4H, $NringCH_2$).

^{13}C NMR (270 MHz, D_2O): δ 174.78 ppm (1C, amide carbonyl), 168.60 ppm (2C, amide carbonyl), 150.31 ppm (1C, aromatic ring), 141.34 ppm (1C, aromatic ring), 123.37 ppm (1C, aromatic ring), 60.00 ppm (2C, ArCH$_2$Nring), 57.51 ppm (2C, acetamide methylene), 56.29 ppm (1C, acetamide methylene), 55.23 ppm (2C, NringCH$_2$), 52.02 ppm (2C, NringCH$_2$);

ESI-MS m/z (%): 378.3 (100%) $[M+H]^+$, 400.2 (34%) $[M+Na]^+$= 416.2 (14%) $[M+K]^+$.

Anal. Calcd for PCTMA^H×1.95 CF_3COOH×0.90 H_2O: C, 39.96; H, 5.05; N, 15.54. Found: C, 40.28; H, 5.11; N, 15.61.

PC3AM^{Pip}: A solution of pyclen (0.25 g, 1.21 mmol), synthesized 2-bromo-1-(piperidin-1-yl)ethanone (0.80 g, 3.88 mmol) and potassium carbonate (1.20 g, 8.68 mmol) in anhydrous acetonitrile was heated to 65 °C with stirring for 3 days. The solution was then filtered and the filtrate evaporated under vacuum. The resulting residue was redissolved water and acetonitrile mixture (50:50 by volume and purified by preparative HPLC using Luna 10u-Prep C18(2) 100A (250×21.20 mm) column and ACN:H2O/TFA was applied as eluent (the TFA was present only in water at 0.005 M concentration). The product was collected by freeze-drying as white fluffy solid. Yield: 0.52 g (58%).

^1H NMR (270 MHz, D_2O): δ 7.92 ppm (dt, 1H, aromatic ring), 7.39 ppm (dd, 2H, aromatic ring), 4.73 ppm (s, 4H, ArCH$_2$Nring), 4.41 ppm (s, 4H, amide methylene), 3.69 ppm (s, 2H, amide methylene), 3.05-3.42 ppm (b, 20H, NringCH$_2$ and piperidine CH$_2$), 1.46-1.53 ppm (b, 18H, piperidine CH$_2$).

^{13}C NMR (270 MHz, D_2O): δ 169.05 ppm (1C, amide carbonyl), 168.71 ppm (2C, amide carbonyl), 158.73 ppm (1C, aromatic ring), 137.52 ppm (1C, aromatic ring), 137.35 ppm (1C, aromatic ring), 121.30 ppm (1C, aromatic ring), 62.44 ppm (2C, ArCH$_2$Nring), 58.10 ppm (2C, acetamide methylene), 55.22 ppm (1C, acetamide methylene), 53.01 ppm (2C, NringCH$_2$), 45.99 ppm (2C, NringCH$_2$) 42.93 42.86 (4C, piperidine ring), 44.44, 43.36 (4C, piperidine ring), 42.93, 42.86 (4C, piperidine ring), 26.24, 25.58, 24.43 (6C, piperidine ring), 25.99, 25.32 24.24 (3C, piperidine ring).

ESI-MS m/z (%): 582.4 (100%) $[M+H]^+$, 604.4 (41%) $[M+Na]^+$= 620.4 (17%) $[M+K]^+$.

Anal. Calcd for PCTMA^{Pip}×1.90 CF_3COOH×0.75 H_2O: C, 52.23; H, 6.70; N, 11.84. Found: C, 52.17; H, 6.61; N, 11.73.

2 Supplementary Table S1. Protonation constants of the hexa-, hepta- and octadentate ligands (I=0.15 M NaCl, 25 °C) compared to the data available in the literature.

Ligand	I	log K_1^H	log K_2^H	log K_3^H	log K_4^H	log K_5^H	log K_6^H	Σ logK_2^H
**DO3A**	**0.15 M NaCl**	**10.07(5)**	**8.93(6)**	**4.43(9)**	**4.11(7)**	**1.88(7)**	**–**	**19.00**
**DO3A^n**	0.1 M NMe$_4$Cl	11.59	9.24	4.43	3.48	–	–	20.83

**DO3A**[o]	0.5 M KNO_3	11.96	9.66	4.23	3.51	–	–	21.62
**DO3A**[p]	0,1 M NaCl	10.51	9.08	4.36	–	–	–	19.59
**DO3A**[q]	0,1 M KCl	11.55	9.15	4.48	–	–	–	20.70
**DO3AM^{H}**	**0.15 M NaCl**	**9.40(5)**	**6.28(8)**	**–**	**–**	**–**	**–**	**15.68**
**DO3P**	**0.15 M NaCl**	**12.55(2)**	**11.37(1)**	**8.57(2)**	**7.02(2)**	**5.36(2)**	**1.84(2)**	**23.92**
**DO3P** [l]	0.1 M $TBANO_3$	13.24	11.3	8.47	7.1	5.3	n.d.	24.54
**DO3P** [m]	0.1 M KCl	12.9	11.4	8.69	7.09	5.53	1.42	24.3
**ODO3A**	**0.15 M NaCl**	**8.74(2)**	**7.58(2)**	**3.99(3)**	**2.39(3)**	**–**	**–**	**16.32**
**ODO3A** [r]	0.1 M $NMe_4(NO_3)$	11.61	7.70	4.05	2.77	–	–	19.31
**PCTA**	**0.15 M NaCl**	**9.97(3)**	**6.73(5)**	**3.22(6)**	**1.40(9)**	**–**	**–**	**16.70**
**PCTA** [g]	0.1 M $NMe_4(NO_3)$	10.90	7.11	3.88	2.27	–	—	18.01
**PCTA** [h]	1.0 M KCl	11.36	7.35	3.83	2.12	1.29		18.71
**PCTA** [i]	0.1 M KCl	10.73	7.52	4.2	2.4	–	–	18.25
**PC3AM^{H}**	**0.15 M NaCl**	**8.76(3)**	**4.10(4)**	**–**	**–**	**–**	**–**	**12.86**
**PC3AM^{Gly}**	**0.15 M NaCl**	**8.85(1)**	**4.55(1)**	**3.81(1)**	**3.21(1)**	**2.80(1)**	**1.38(1)**	**13.40**
**PC3AM^{Gly}** [j]	1.0 M KCl	9.35	4.47	3.73	3.20	2.72	–	13.82
**PC3AM^{Pip}**	**0.15 M NaCl**	**8.74(1)**	**5.77(2)**	**1.42(9)**	**–**	**–**	**–**	**14.51**
**DOTA**	0.15 M NaCl	11.41	9.83	4.38	4.63	1.92	1.58	21.24
**DOTA**[a]	0.1 M NMe4Cl	11.74	9.67	4.68	4.11	2.37	–	21.41
**DOTA**[b]	0.1 M NMe4Cl	12.6	9.70	4.50	4.14	2.32	–	22.30
**DOTA**[c]	0.1 M NMe4Cl	11.9	9.72	4.60	4.13	2.36	–	21.62
**DOTMA**	0.15 M NaCl	11.72	9.06	4.74	5.59	1.92	–	20.78
**DOTMA** [k]	1.0 M KCl	12.59	9.28	4.66	5.78	1.84	–	21.87
**DOTAM**	**0.15 M NaCl**	**7.31(1)**	**6.07(1)**	**–**	**–**	**–**	**–**	**13.38**
**DOTP**	**0.15 M NaCl**	**13.6(2)**[a]	**12.23(3)**	**8.63(5)**	**7.45(4)**	**5.84(5)**	**5.02(5)**	**25.83**
**DOTP** [d]	0.1 M $NMe_4(NO_3)$	13.7	12.2	9.28	8.09	6.12	5.22	25.9
**DOTP** [e]	0.15 M NaCl	10.9	9.2	8.1	6.3	5.4	1.8 and 1.3 pK_7^H	20.1
**DOTP** [f]	0.1 M NMe_4Cl	12.6	9.3	8.0	6.0	5.2	–	21.9

***trans*-DO2A**	0.15 M NaCl	11.69	9.75	3.97	2.68	–	–	21.44
***cis*-DO2A**	0.15 M NaCl	11.44	9.51	4.14	1.55	–	–	20.95
***cis*-DO2AM^{Me2}.**	0.1 M KCl	10.14	8.38	–	–	–	–	18.52
***cis*-Bz2DO2AM^{Me2}.**	0.1 M KCl	11.11	8.22	–	–	–	–	19.33
***cis*-DO2AM^{Bz2}.**	0.1 M KCl	9.62	6.90	–	–	–	–	16.52

[a] Antonio Bianchi, Luisella Calabi, Claudia Giorgi, Pietro Losi, Palma Mariani, Paola Paoli, Patrizia Rossi, Barbara Valtancoli, Mario Virtuani. 2000. Thermodynamic and structural properties of Gd3+ complexes with functionalized macrocyclic ligands based upon 1,4,7,10- tetraazacyclododecane; J. Chem. Soc. Dalton Trans. 5, 697-705, DOI: 10.1039/A909098C.

[b] László Burai, István Fábián, Róbert Király, Erika Szilágyi, Ernö Brücher. 1998. Equilibrium and kinetic studies on the formation of the lanthanide(III) complexes, [Ce(dota)]– and [Yb(dota)]– (H4dota = 1,4,7,10-tetraazacyclododecane-1,4,7,10-tetraacetic acid), J. Chem. Soc., Dalton Trans., 2, 243-248; DOI: 10.1039/A705158A

[c] Giorgio Anderegg, Francoise Arnaud-Neu, Rita Delgado, Judith Felcman, Konstantin Popov. 2005. Critical evaluation of stability constants of metal complexes of complexones for biomedical and environmental applications (IUPAC Technical Report). Pure Appl. Chem. 77, 1445–1495. DOI: 10.1351/pac200577081445

[d] Rita Delgado, Liselotte C. Siegfried, Thomas A. Kaden. 1990. Metal Complexes with Macrocyclic Ligands. Part XXXI*. Protonation studies and complexation properties of tetraazamacrocyclic methylenephosphonates with earth ‐ alkali ions. Helv. Chim. Acta 73, 140. DOI: 10.1002/hlca.19900730115

[e, f] C. F. G. C. Geraldes, A. D. Sherry, and W. P. Cacheris. 1989. Synthesis, protonation sequence, and NMR studies of polyazamacrocyclic methylenephosphonates. Inorg. Chem. 28(17), 3336–3341. DOI: 10.1021/ic00316a018

[g] Rita Delgado, Sandra Quintino, Miguel Teixeira, Anjiang Zhang, 1997. Metal complexes of a 12-membered tetraaza macrocycle containingpyridine and N-carboxymethyl groups. J. Chem. Soc., Dalton Trans., 55-64. DOI: 10.1039/A602311H

[h] Gyula Tircsó, Zoltán Kovács, and A. Dean Sherry. 2006. Equilibrium and Formation/Dissociation Kinetics of Some Ln^{III}PCTA Complexes, Inorg. Chem., 45(23), 9269–9280. DOI: 10.1021/ic0608750

[i] Krishan Kumar, and M. F. Tweedle. 1993. Ligand basicity and rigidity control formation of macrocyclic polyamino carboxylate complexes of gadolinium(III); Inorg. Chem., 32(20), 4193–4199. DOI: 10.1021/ic00072a008

[j] Federico A. Rojas‐Quijano, Enikő Tircsóné Benyó, Gyula Tircsó, Ferenc K. Kálmán, Zsolt Baranyai, Silvio Aime, A. Dean Sherry, Zoltán Kovács. 2009. Lanthanide(III) Complexes of Tris(amide) PCTA Derivatives as Potential Bimodal Magnetic Resonance and Optical Imaging Agents. Chem. Eur. J. 15(47), 13188-200. DOI: 10.1002/chem.200901095

[k] Silvio Aime, Mauro Botta, Zoltán Garda, Benjamin E. Kucera, Gyula Tircso, Victor G. Young, and Mark Woods. 2011. Properties, Solution State Behavior, and Crystal Structures of Chelates of DOTMA. Inorg. Chem., 50(17), 7955–7965. DOI: 10.1021/ic2012827

[l] Ute Kreher, Milton T. W. Hearn, Leone Spiccia. 2009. X-Ray Crystal Structure, Acid–Base Properties and Complexation Characteristics of a Methylenephosphonate Derivative of 1,4,7,10-Tetraazacyclododecane, Australian Journal of Chemistry 62(12) 1583-1592. Australian Journal of Chemistry 62(12) 1583-1592. https://doi.org/10.1071/CH09337

[m] X. Sun, M. Wuest, Z. Kovacs, A. D. Sherry. 2003. In vivo behavior of copper-64-labeled methanephosphonate tetraaza macrocyclic ligands. J. Biol. Inorg. Chem. 8, 217. DOI: 10.1007/S00775-002-0408-5

[n, p, q] Krishan Kumar, C. Allen Chang, L. C. Francesconi, D. D. Dischino, M. F. Malley, J. Z Gougoutas, and M. F. Tweedle. 1994. Synthesis, Stability, and Structure of Gadolinium(III) and Yttrium(III) Macrocyclic Poly(amino carboxylates). Inorg. Chem., 33(16), 3567–3575. DOI: 10.1021/ic00094a021

[o] Hui‐Zhi Cai, Thomas A. Kaden. 1994. Metal complexes with macrocyclic ligands. Part XXXVI*. Thermodynamic and kinetic studies of bivalent and trivalent metal ions with 1,4,7,10‐tetraazacyclododecane‐1,4,7‐triacetic acid. Helv. Chim. Acta, 77, 383. DOI: 10.1002/hlca.1994077013.

[r] M. T. S. Amorim, Rita Delgado, J. J. R. Fraústo da Silva, M. Cândida, T. A. Vaz, M. Fernanda Vilhena. 1988. Metal complexes of 1-oxa-4,7,10-triazacyclododecane-N,N′,N″-triacetic acid. Talanta, 35(9) 741-745. DOI: 10.1016/0039-9140(88)80175-9

3 Supplementary Figures

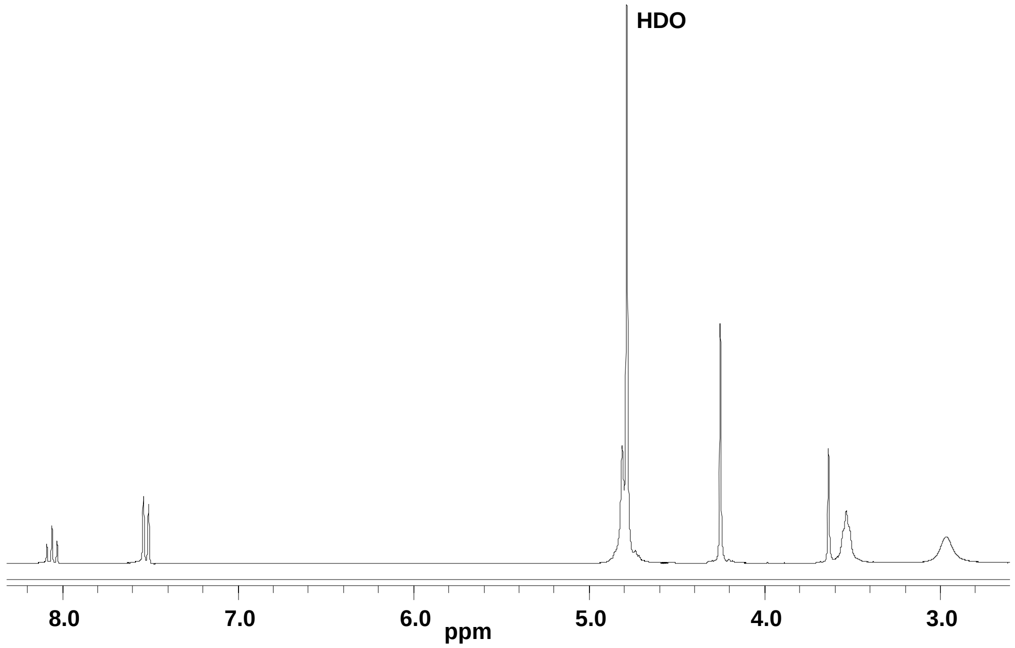

Supplementary Figure 1. ^{1}H-NMR of the PC3AM^H recorded in D_2O at 270 MHz at 25 ^oC.

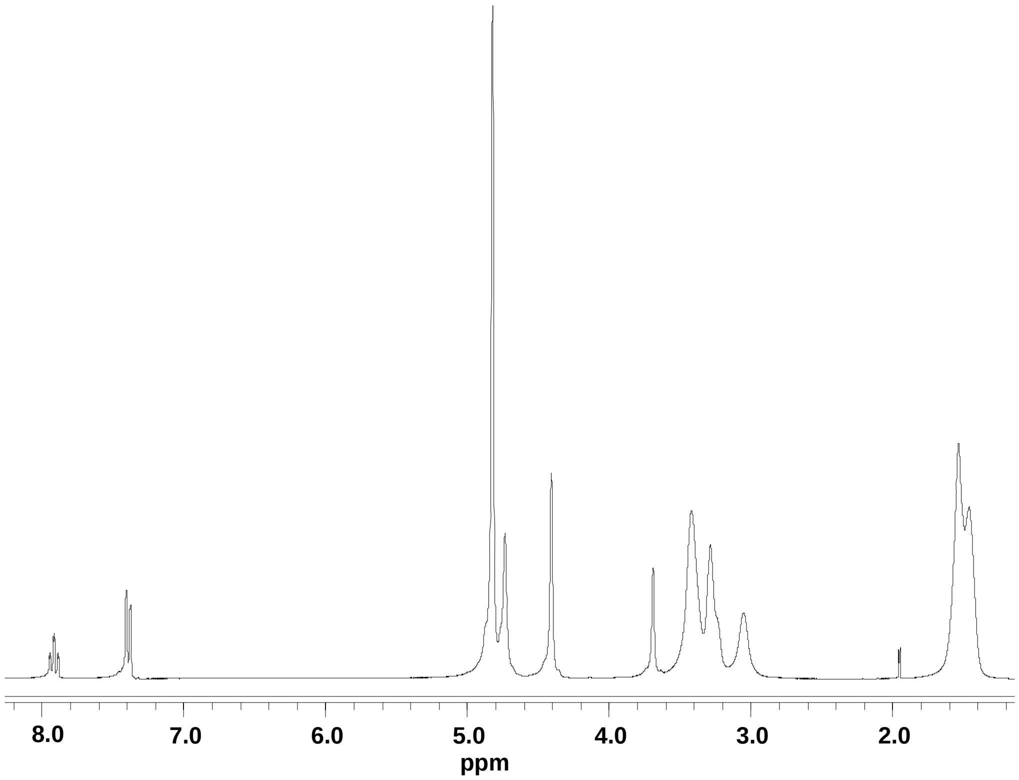

Supplementary Figure 2. ^{1}H-NMR of the PC3AM^{Pip} recorded in D_2O at 270 MHz at 25 °C.

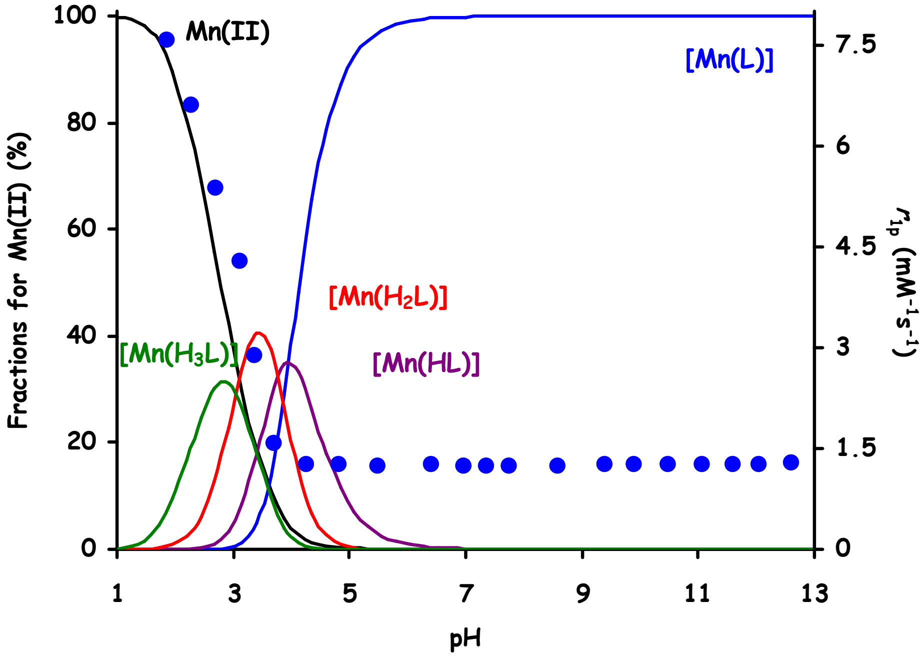

Supplementary Figure 3. Speciation distribution curves of $[Mn(DOTA)]^{2-}$ complex with the pH dependence of its relaxivity (I=0.1 M KCl, T=25 °C, 20 MHz).

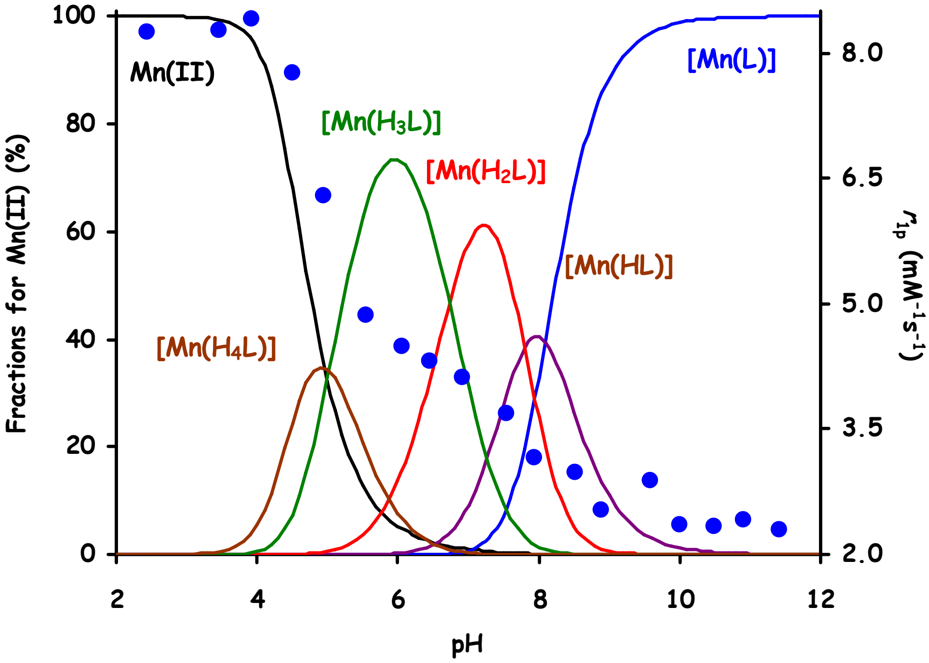

Supplementary Figure 4. The speciation distribution of $[Mn(DOTP)]^{6-}$ complex with the pH dependence of its relaxivity (*I*=0.15 M NaCl, *T*=25 °C, 20 MHz).

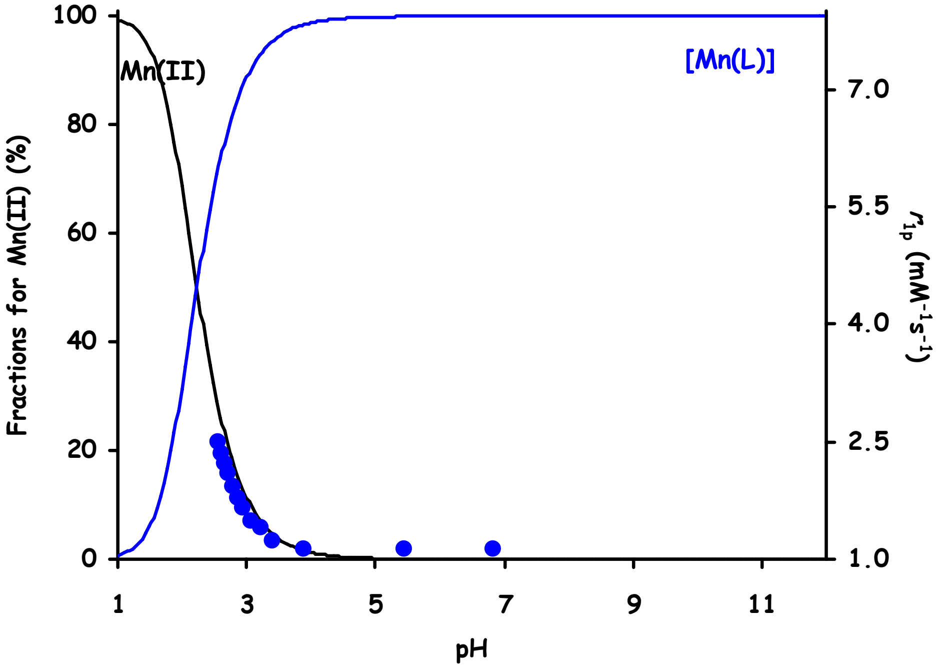

Supplementary Figure 5. Relaxivity (r_{1p}) measured for "batch" samples of $[Mn(DOTAM)]^{2+}$ complex as a function of pH and species distribution curves obtained from the analysis of the relaxivity data (*T*=25 °C, *I*=0.15 M NaCl, 20 MHz).

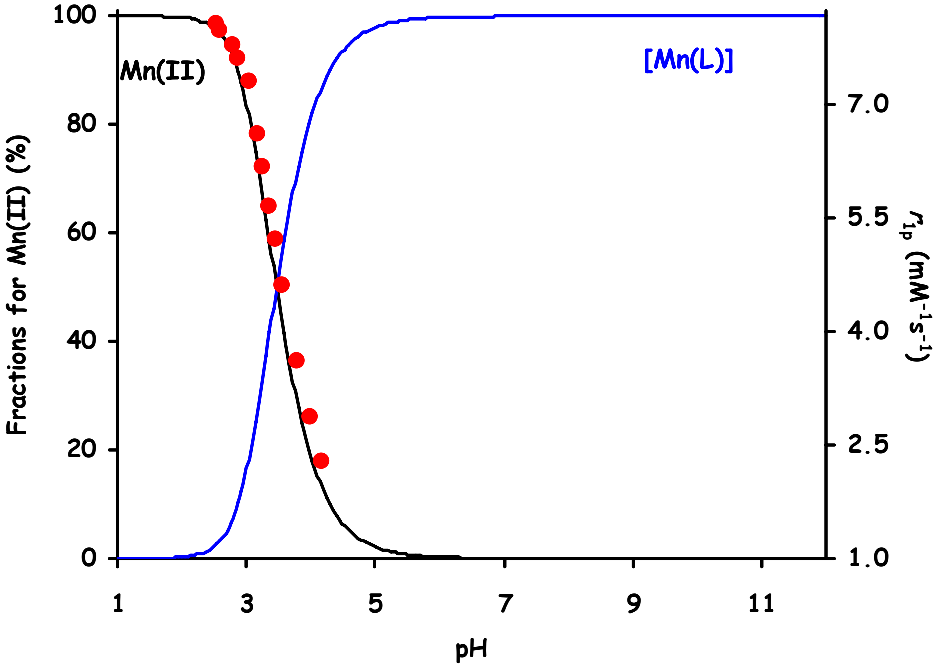

Supplementary Figure 6. Relaxivity (r_{1p}) measured for "batch" samples of $[Mn(DO3AM^{H})]^{2+}$ complex as a function of pH and species distribution curves obtained from the analysis of the relaxivity data (T=25 ºC, I=0.15 M NaCl, 20 MHz).

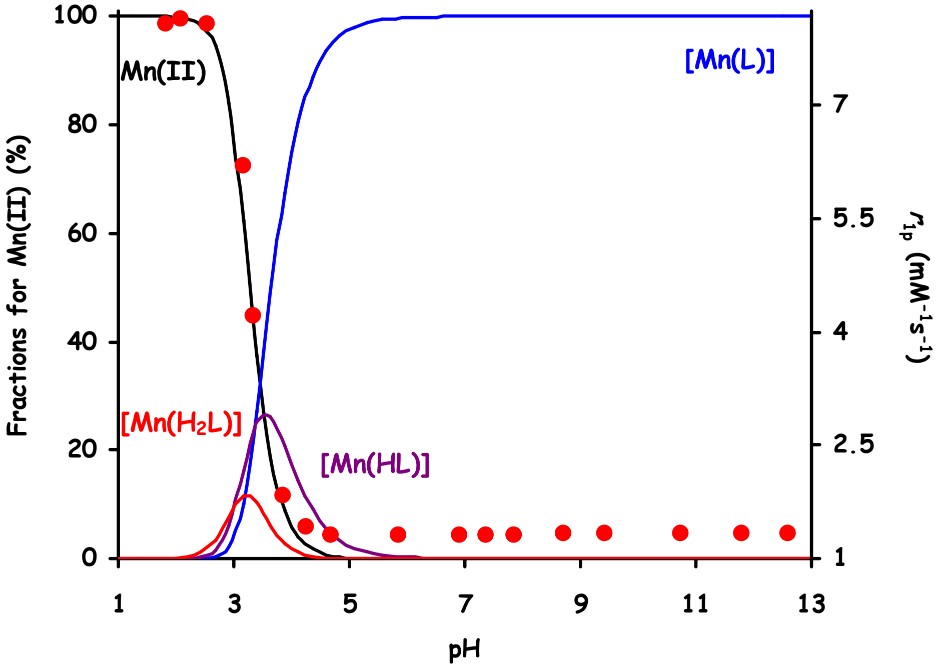

Supplementary Figure 7. Speciation distribution curves of $[Mn(DO3A)]^{-}$ complex with the pH dependence of its relaxivity (I=0.1 M KCl, T=25 °C, 20 MHz).

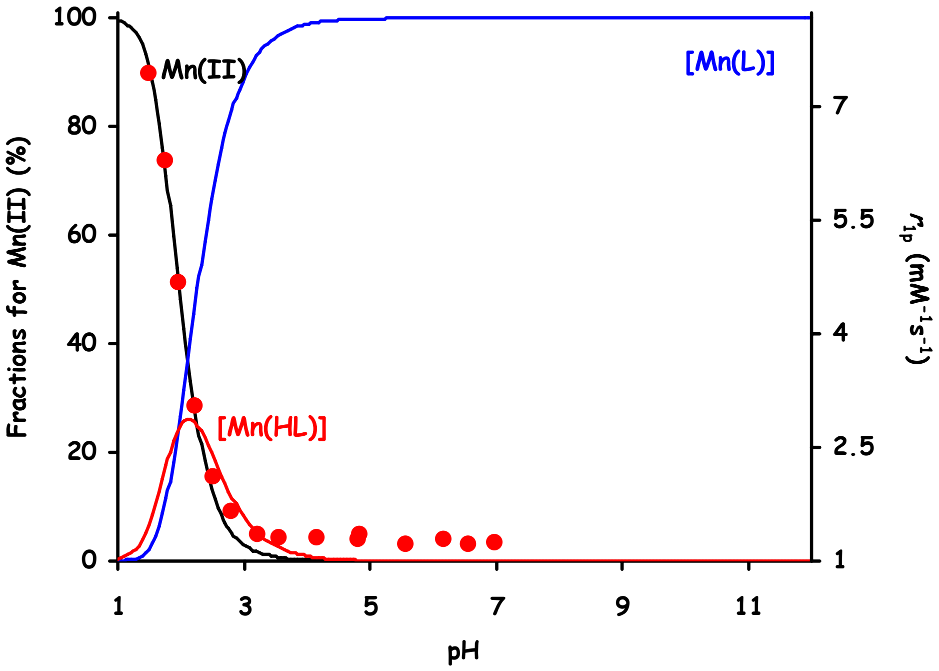

Supplementary Figure 8. Speciation distribution curves of $[Mn(PCTA)]^{-}$ complex co-plotted with the pH dependence of its relaxivity (I=0.15 M NaCl, T=25 °C, 20 MHz).

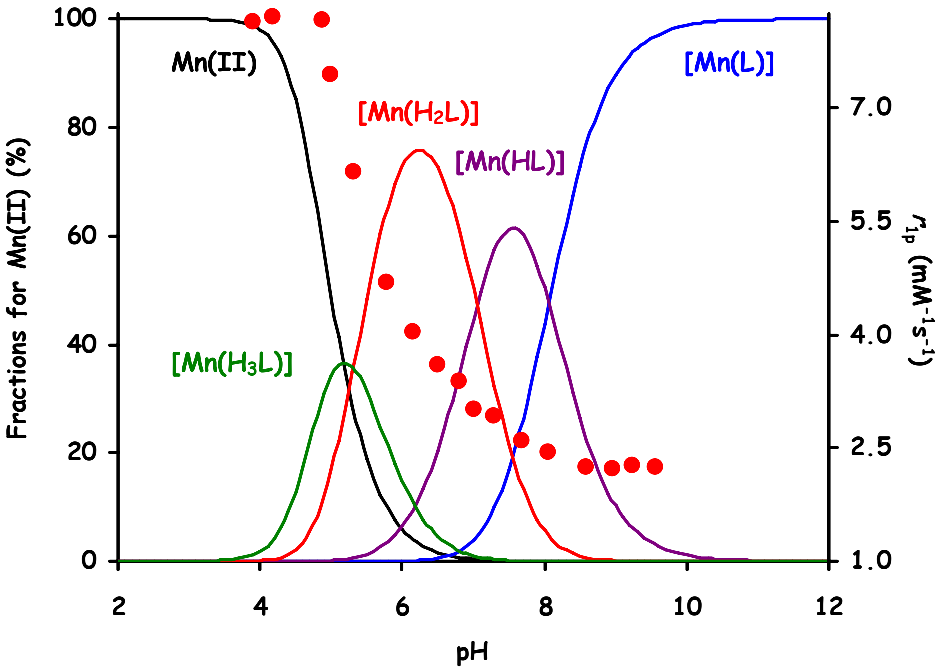

Supplementary Figure 9. Speciation distribution curves of $[Mn(DO3P)]^{4-}$ complex with the pH dependence of its relaxivity (I=0.15 M NaCl, T=25 °C, 20 MHz).

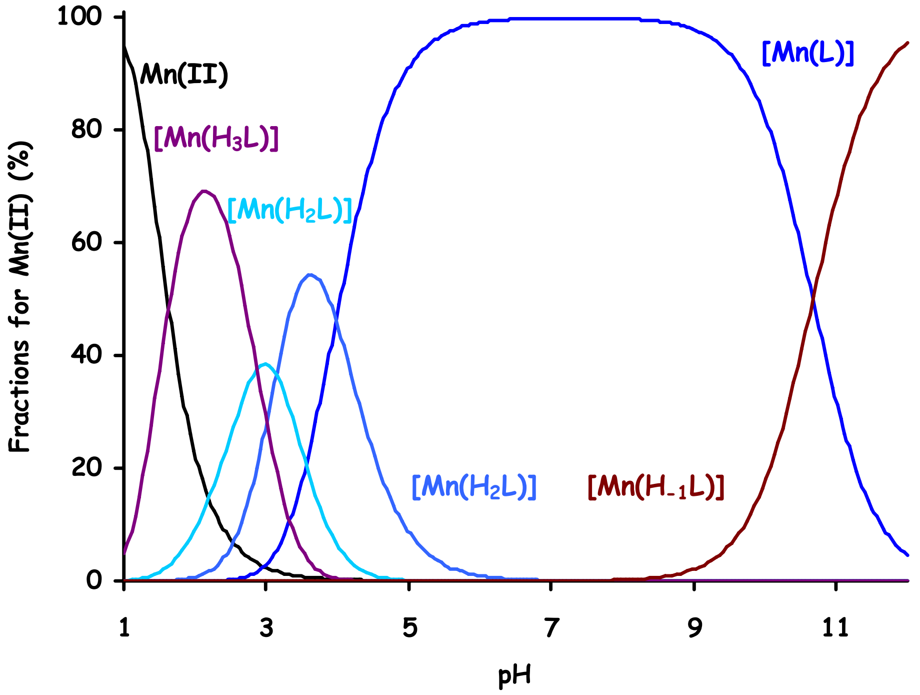

Supplementary Figure 10. Speciation distribution curves of $[Mn(PC3AMGly)]^{-}$ complex (c_L=c_{Mn2+}=0.002 M, I=0.15 M NaCl and T=25 °C).

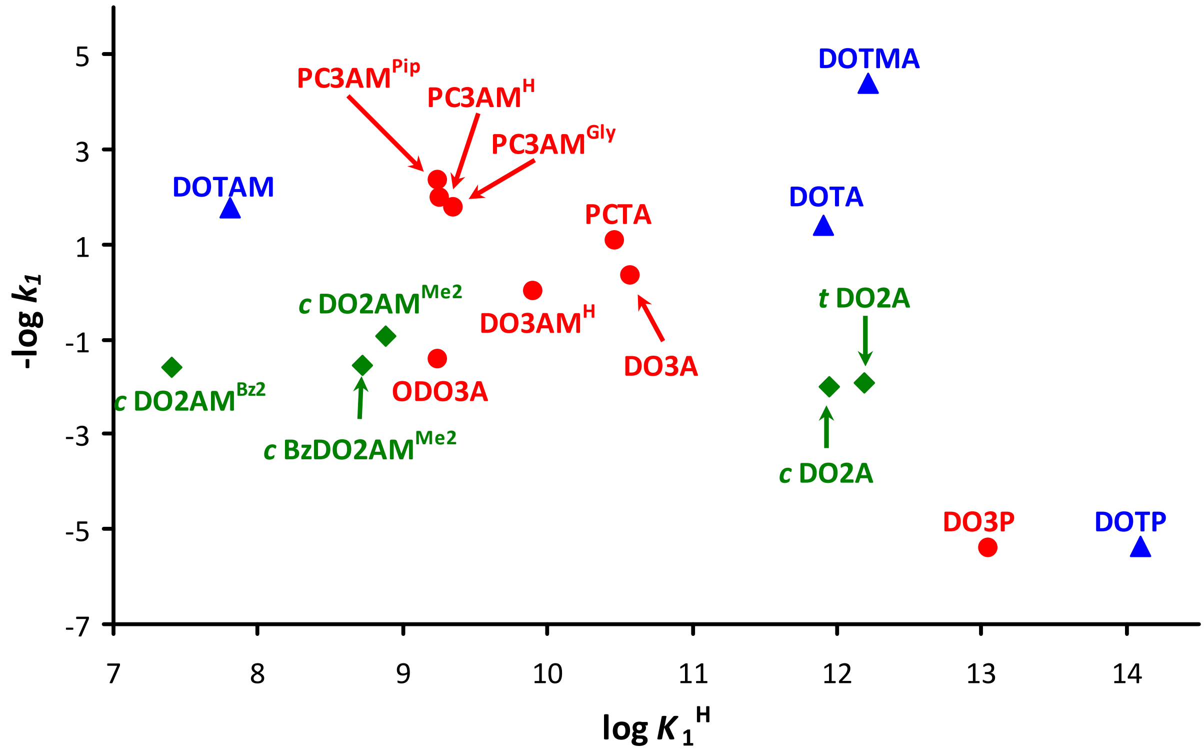

Supplementary Figure 11. Plot of – log k_1 values (k_1 is the rate constant of the acid catalyzed dissociation) as a function of log log K_1^H for derivatives of 12-membered macrocycles (I=0.15 M NaCl and 25 °C). Disubstituted ligands are shown in green, trisubstituted derivatives are in red (for these the -log k_1 = -1.61×log K_1^H + 15.64 with R^2 = 0.69) and tetrasubstituted derivatives are in blue.

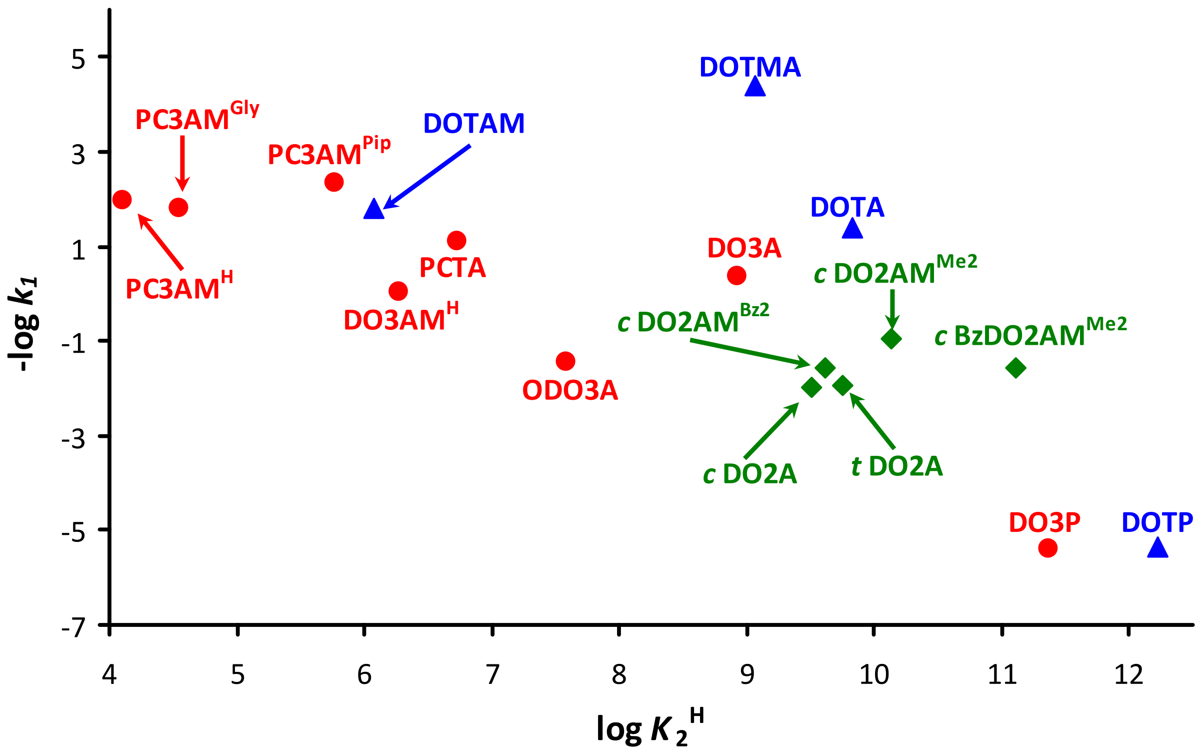

Supplementary Figure 12. Plot of – log k_1 values (k_1 is the rate constant of the acid catalyzed dissociation) as a function of log log K_2^H for derivatives of 12-membered macrocycles (I=0.15 M NaCl and 25 °C). Disubstituted ligands are shown in green, trisubstituted derivatives are in red (-log k_1 = -0.93×log K_2^H + 6.53 with R^2 = 0.77) and tetrasubstituted derivatives are in blue.

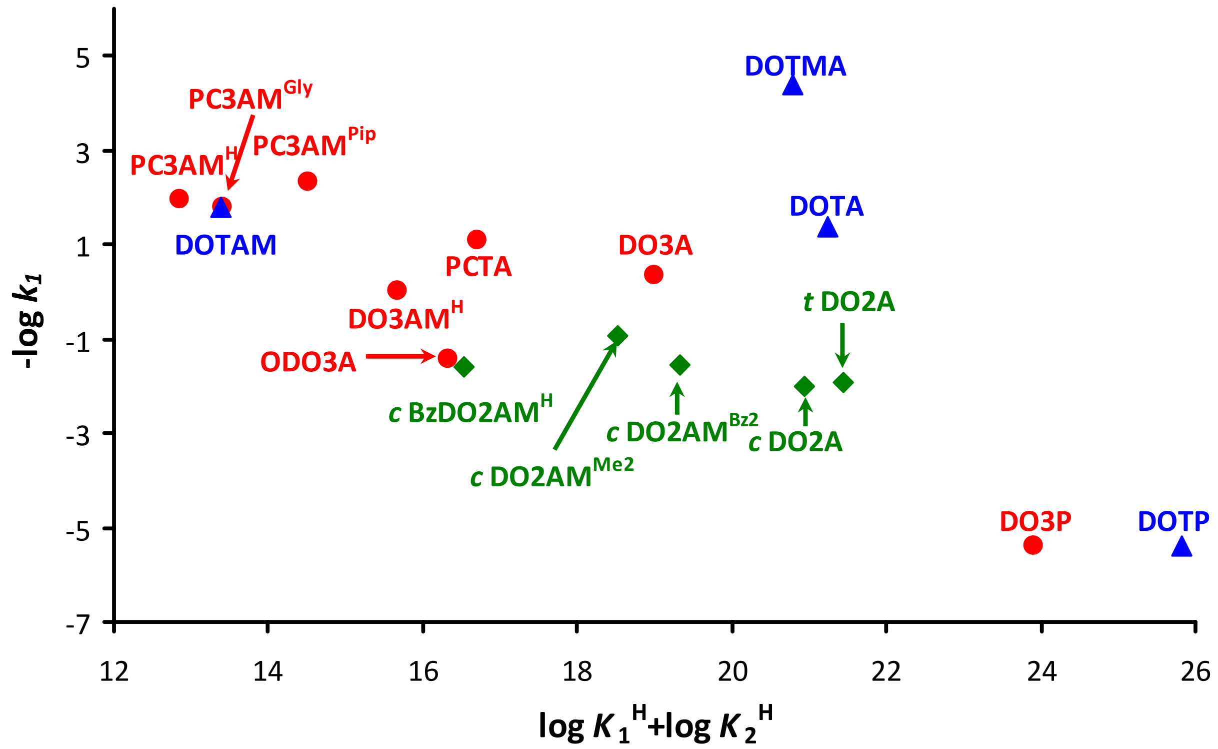

Supplementary Figure 13. Plot of – log k_1 values (k_1 is the rate constant of the acid catalyzed dissociation) as a function of log K_1^H+ log K_2^H for derivatives of 12-membered macrocycles (I=0.15 M NaCl and T=25 °C). Disubstituted ligands are shown in green, trisubstituted derivatives are in red (-log k_1 = -0.63×(log K_1^H + log K_2^H) + 10.52 with R^2 = 0.79) and tetrasubstituted derivatives are in blue.

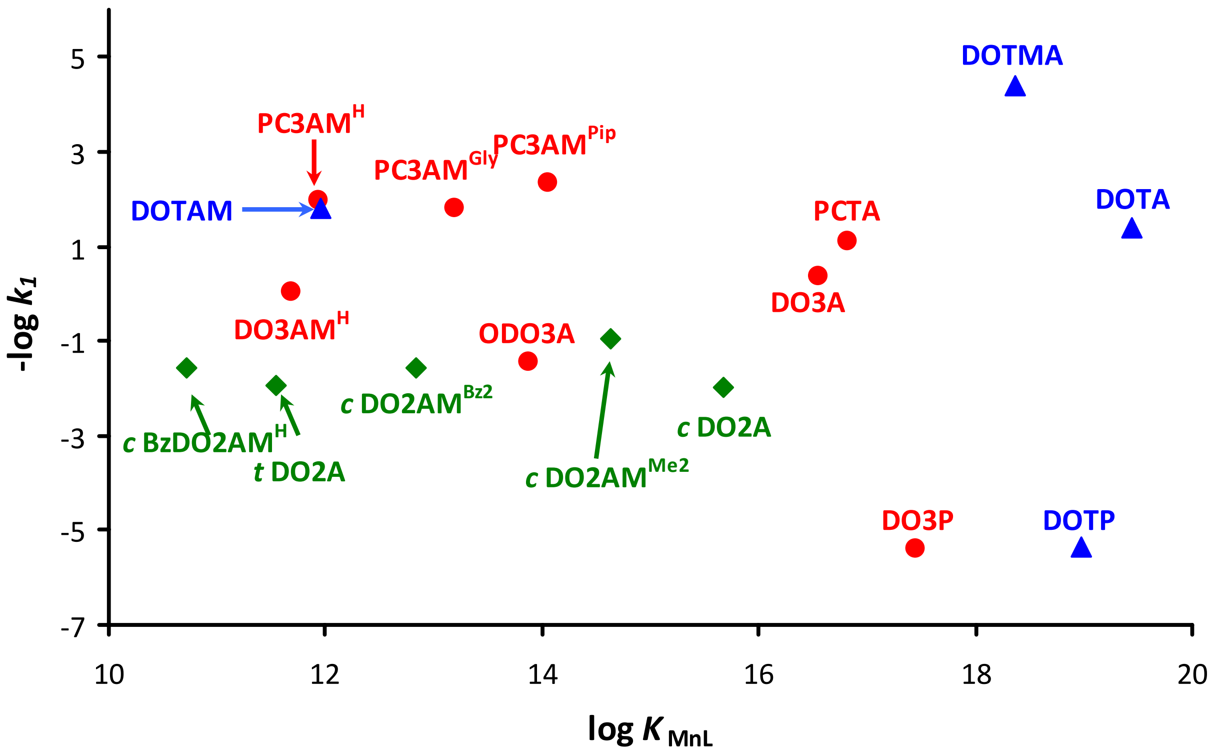

Supplementary Figure 14. Plot of – log k_1 values (k_1 is the rate constant of the acid catalyzed dissociation) as a function of log $K_{[Mn(L)]}$ for derivatives of 12-membered macrocycles (I=0.15 M NaCl and T=25 °C). Disubstituted ligands are shown in green, trisubstituted derivatives are in red (-log k_1 = -0.58 × log K_{MnL} + 8.42 with R^2 = 0.26) and tetrasubstituted derivatives are in blue.

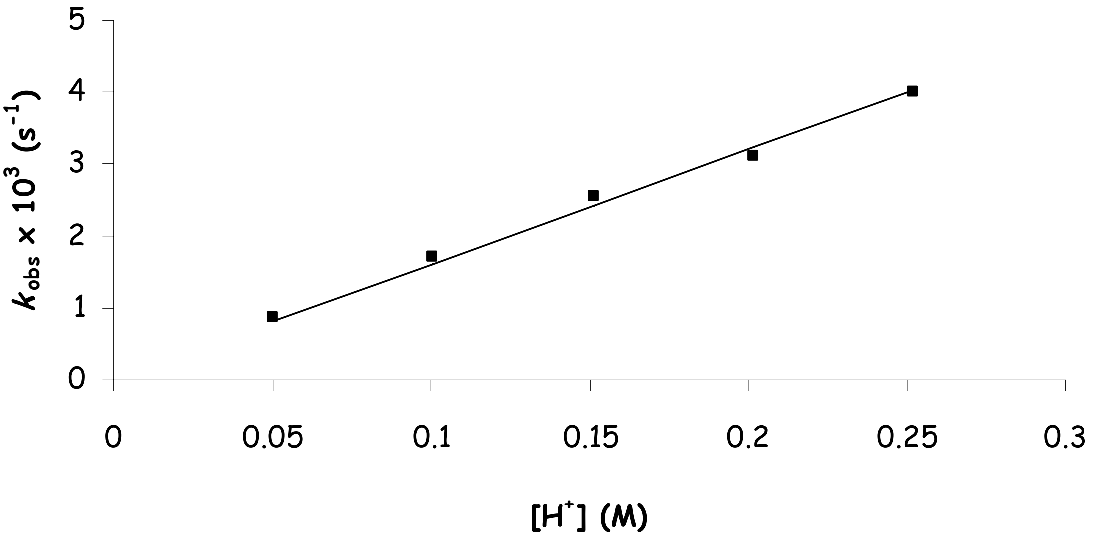

Supplementary Figure 15. Dependence of the pseudo-first-order rate constants (k_{obs}) on the concentration of H^+ ion for the $[Mn(DOTAM)]^{2+}$ complex (T=25 °C).

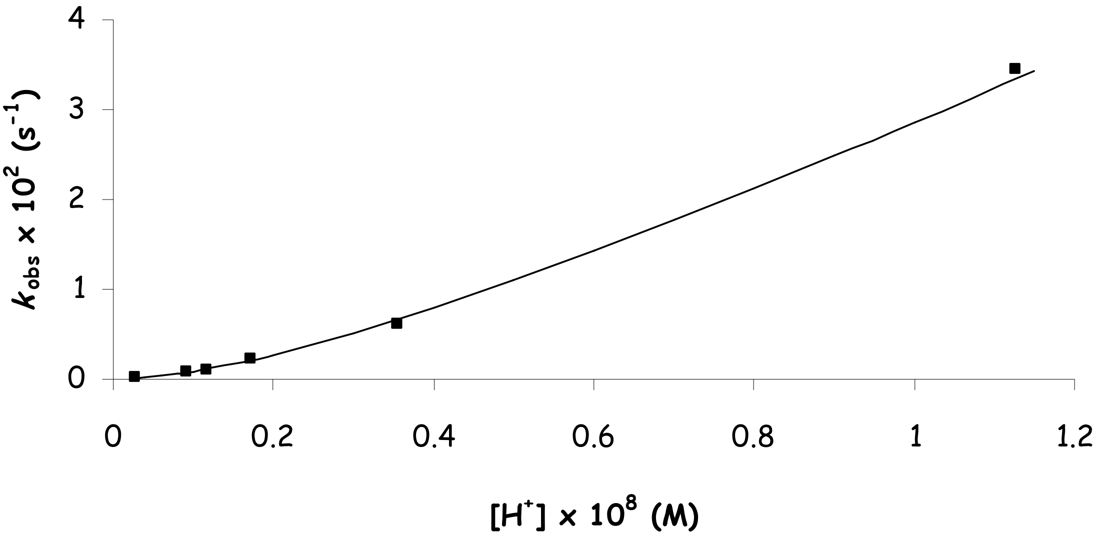

Supplementary Figure 16. Dependence of the pseudo-first-order rate constants (k_{obs}) on the concentration of H^+ ion for the $[Mn(DOTP)]^{6-}$ complex (T=25 °C).

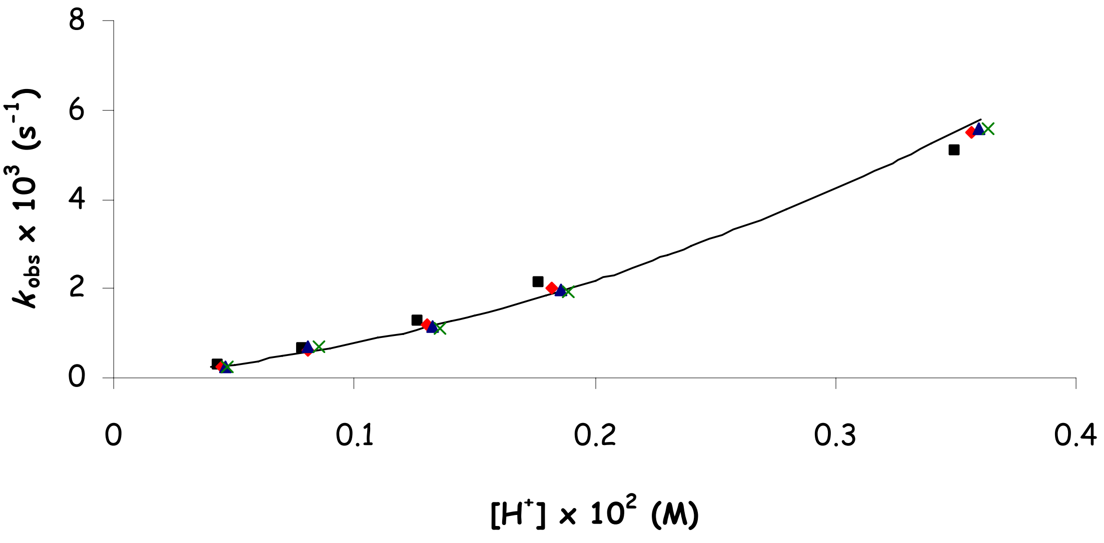

Supplementary Figure 17. Dependence of the pseudo-first-order rate constants (k_{obs}) on the concentration of the Cu^{2+} and H^+ ions for the $[Mn(DO3A)]^-$ complex. The excess of the exchanging metal ion was ×12(■), ×24(♦), ×32(▲) and ×41(×) folds (T=25 °C).

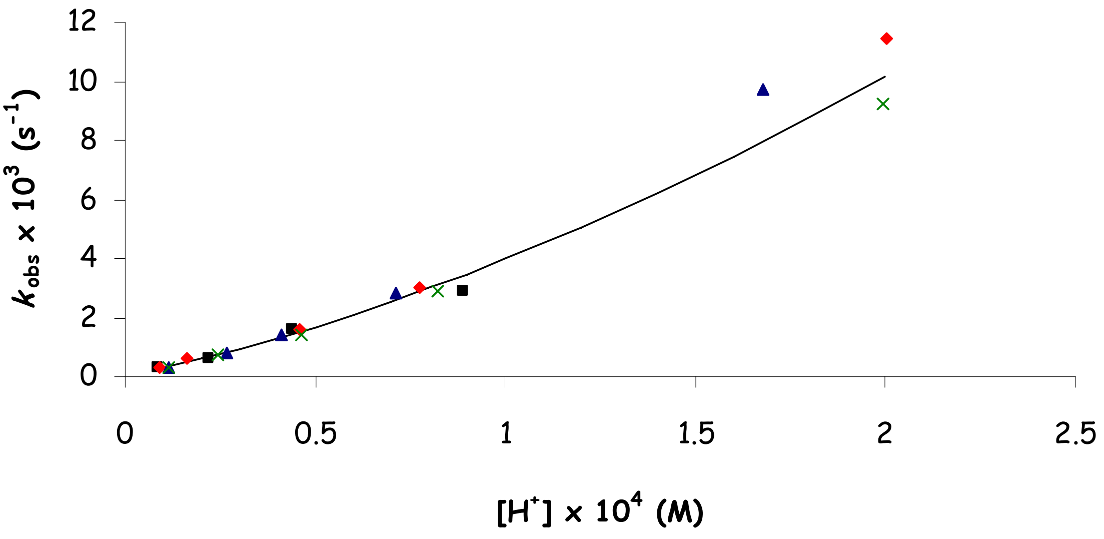

Supplementary Figure 18. Dependence of the pseudo-first-order rate constants (k_{obs}) on the concentration of the Cu^{2+} and H^+ ions for the $[Mn(ODO3A)]^-$ complex. The excess of the exchanging metal ion was ×10(■), ×20(♦), ×30(▲) and ×40(×) folds (T=25 °C).

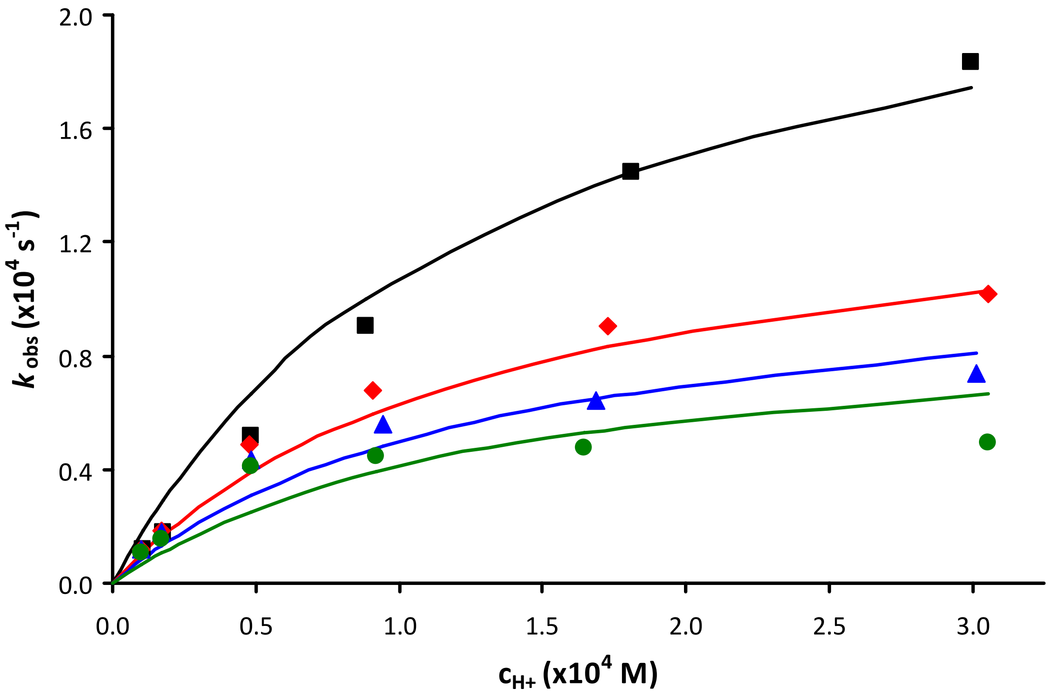

Supplementary Figure 19. Dependence of the pseudo-first-order rate constants (k_{obs}) on the concentration of H^+ and Cu^{2+} ions for the $[Mn(DO3AM^H)]^{2+}$ complex (T=25 °C). The excess of the exchanging metal ion was ×10(■), ×20(♦), ×30(▲) and ×40(●) folds.

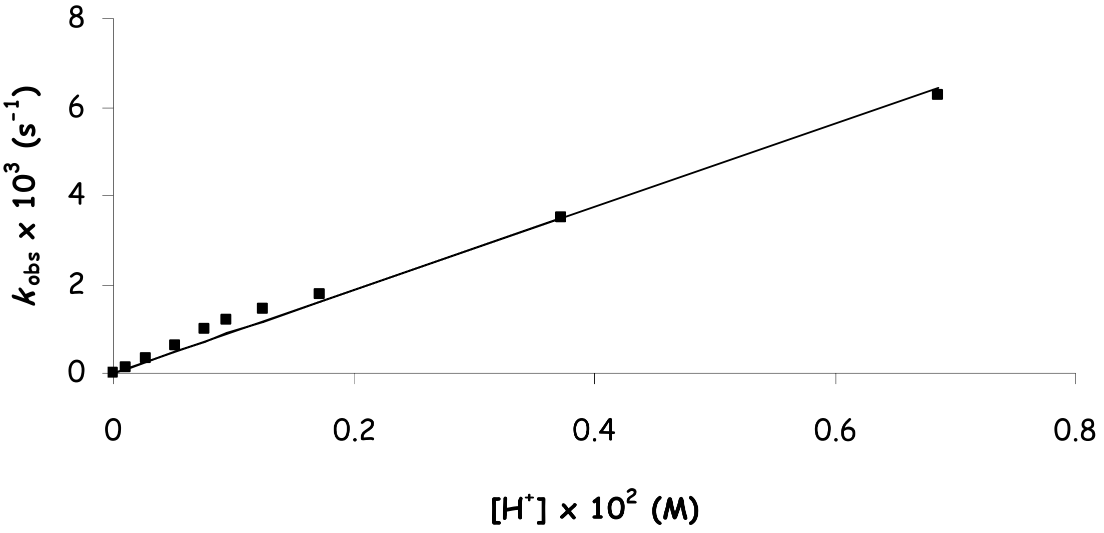

Supplementary Figure 20. Dependence of the pseudo-first-order rate constants (k_{obs}) on the concentration of H^+ ion for the $[Mn(DO3AM^H)]^{2+}$ complex (T=25 °C).

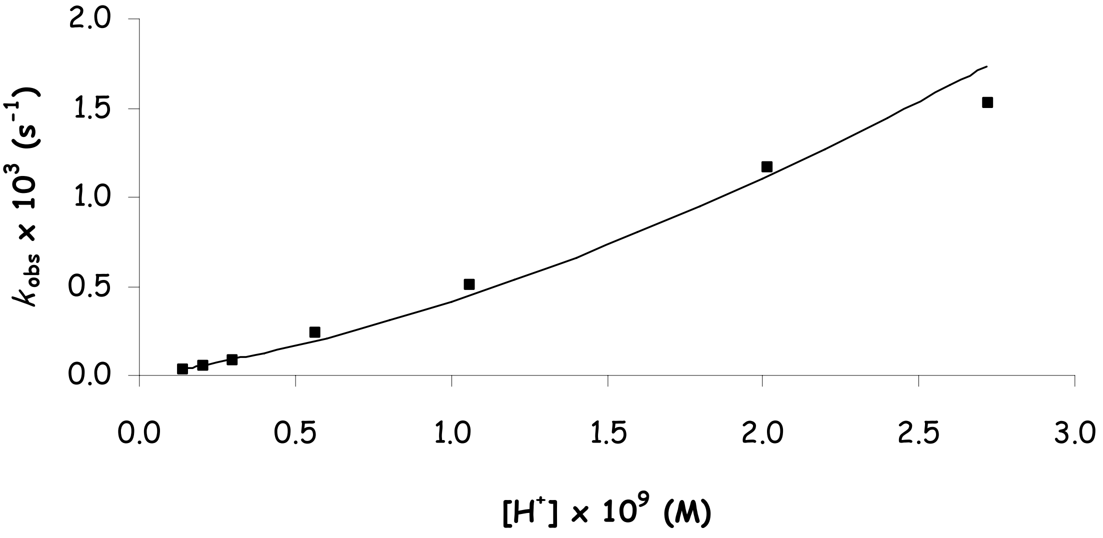

Supplementary Figure 21. Dependence of the pseudo-first-order rate constants (k_{obs}) on the concentration of H^+ ion for the $[Mn(DO3P)]^{4-}$ complex (T=25 °C).
